# Neurological disease in xeroderma pigmentosum: prospective cohort study of its features and progression

**DOI:** 10.1093/brain/awad266

**Published:** 2023-12-01

**Authors:** Hector Garcia-Moreno, Douglas R Langbehn, Adesoji Abiona, Isabel Garrood, Zofia Fleszar, Marta Antonia Manes, Ana M Susana Morley, Emma Craythorne, Shehla Mohammed, Tanya Henshaw, Sally Turner, Harsha Naik, Istvan Bodi, Robert P E Sarkany, Hiva Fassihi, Alan R Lehmann, Paola Giunti

**Affiliations:** Ataxia Centre, Department of Clinical and Movement Neurosciences, UCL Queen Square Institute of Neurology, London WC1N 3BG, UK; Department of Psychiatry, University of Iowa Carver College of Medicine, Iowa City, IA 52242, USA; UK National Xeroderma Pigmentosum Service, Guy’s and St Thomas’ NHS Foundation Trust, London SE1 7EH, UK; UK National Xeroderma Pigmentosum Service, Guy’s and St Thomas’ NHS Foundation Trust, London SE1 7EH, UK; Ataxia Centre, Department of Clinical and Movement Neurosciences, UCL Queen Square Institute of Neurology, London WC1N 3BG, UK; Ataxia Centre, Department of Clinical and Movement Neurosciences, UCL Queen Square Institute of Neurology, London WC1N 3BG, UK; UK National Xeroderma Pigmentosum Service, Guy’s and St Thomas’ NHS Foundation Trust, London SE1 7EH, UK; Department of Ophthalmology, Guy’s and St Thomas’ NHS Foundation Trust, London SE1 7EH, UK; UK National Xeroderma Pigmentosum Service, Guy’s and St Thomas’ NHS Foundation Trust, London SE1 7EH, UK; UK National Xeroderma Pigmentosum Service, Guy’s and St Thomas’ NHS Foundation Trust, London SE1 7EH, UK; UK National Xeroderma Pigmentosum Service, Guy’s and St Thomas’ NHS Foundation Trust, London SE1 7EH, UK; UK National Xeroderma Pigmentosum Service, Guy’s and St Thomas’ NHS Foundation Trust, London SE1 7EH, UK; UK National Xeroderma Pigmentosum Service, Guy’s and St Thomas’ NHS Foundation Trust, London SE1 7EH, UK; Clinical Neuropathology, Academic Neuroscience Building, King’s College Hospital, London SE5 9RS, UK; UK National Xeroderma Pigmentosum Service, Guy’s and St Thomas’ NHS Foundation Trust, London SE1 7EH, UK; UK National Xeroderma Pigmentosum Service, Guy’s and St Thomas’ NHS Foundation Trust, London SE1 7EH, UK; UK National Xeroderma Pigmentosum Service, Guy’s and St Thomas’ NHS Foundation Trust, London SE1 7EH, UK; Genome Damage and Stability Centre, School of Life Sciences, University of Sussex, Falmer, Brighton BN1 9RQ, UK; Ataxia Centre, Department of Clinical and Movement Neurosciences, UCL Queen Square Institute of Neurology, London WC1N 3BG, UK; UK National Xeroderma Pigmentosum Service, Guy’s and St Thomas’ NHS Foundation Trust, London SE1 7EH, UK

**Keywords:** DNA repair, nucleotide excision repair, neurocutaneous syndromes, cerebellar ataxia, Huntington’s disease-like phenotypes, biomarkers

## Abstract

Xeroderma pigmentosum (XP) results from biallelic mutations in any of eight genes involved in DNA repair systems, thus defining eight different genotypes (XPA, XPB, XPC, XPD, XPE, XPF, XPG and XP variant or XPV). In addition to cutaneous and ophthalmological features, some patients present with XP neurological disease. It is unknown whether the different neurological signs and their progression differ among groups. Therefore, we aim to characterize the XP neurological disease and its evolution in the heterogeneous UK XP cohort.

Patients with XP were followed in the UK National XP Service, from 2009 to 2021. Age of onset for different events was recorded. Cerebellar ataxia and additional neurological signs and symptoms were rated with the Scale for the Assessment and Rating of Ataxia (SARA), the Inventory of Non-Ataxia Signs (INAS) and the Activities of Daily Living questionnaire (ADL). Patients’ mutations received scores based on their predicted effects. Data from available ancillary tests were collected.

Ninety-three XP patients were recruited. Thirty-six (38.7%) reported neurological symptoms, especially in the XPA, XPD and XPG groups, with early-onset and late-onset forms, and typically appearing after cutaneous and ophthalmological symptoms. XPA, XPD and XPG patients showed higher SARA scores compared to XPC, XPE and XPV. SARA total scores significantly increased over time in XPD (0.91 points/year, 95% confidence interval: 0.61, 1.21) and XPA (0.63 points/year, 95% confidence interval: 0.38, 0.89). Hyporeflexia, hypopallesthaesia, upper motor neuron signs, chorea, dystonia, oculomotor signs and cognitive impairment were frequent findings in XPA, XPD and XPG. Cerebellar and global brain atrophy, axonal sensory and sensorimotor neuropathies, and sensorineural hearing loss were common findings in patients. Some XPC, XPE and XPV cases presented with abnormalities on examination and/or ancillary tests, suggesting underlying neurological involvement. More severe mutations were associated with a faster progression in SARA total score in XPA (0.40 points/year per 1-unit increase in severity score) and XPD (0.60 points/year per 1-unit increase), and in ADL total score in XPA (0.35 points/year per 1-unit increase).

Symptomatic and asymptomatic forms of neurological disease are frequent in XP patients, and neurological symptoms can be an important cause of disability. Typically, the neurological disease will be preceded by cutaneous and ophthalmological features, and these should be actively searched in patients with idiopathic late-onset neurological syndromes. Scales assessing cerebellar function, especially walking and speech, and disability can show progression in some of the groups. Mutation severity can be used as a prognostic biomarker for stratification purposes in clinical trials.

## Teaser

Garcia-Moreno *et al.* characterize the neurological phenotype of patients with different genetic forms of xeroderma pigmentosum (XP). In XPA and XPD, ataxia scores were found to worsen over time, with faster progression in patients with severe mutations. Subclinical neurological features were observed in XPC, XPE and XPV.

## Introduction

Xeroderma pigmentosum (XP) is a group of multisystemic autosomal recessive conditions secondary to deficits in DNA repair systems.^1-3^ XP can result from variants in any of eight genes, thus defining eight complementation groups: XPA (*XPA*, OMIM number: 611153), XPB (*ERCC3*, OMIM number: 133510), XPC (*XPC*, OMIM number: 613208), XPD (*ERCC2*, OMIM number: 126340), XPE (*DDB2,* OMIM number: 600811), XPF (*ERCC4*, OMIM number 133520), XPG (*ERCC5*, OMIM number: 133530) and XP variant or XPV (*POLH*, OMIM number: 603968). Groups from XPA to XPG present with a defect in the nucleotide excision repair (NER) pathway (reviewed in Lehmann *et al*.^3^). This system repairs bulky helix-distorting DNA lesions caused by ultraviolet radiation (UVR) or chemical carcinogens (reviewed in Lehmann *et al*.^3^ and Jeppesen *et al*.^4^). DNA damage is recognized by two NER sub-pathways, depending on its location. In transcription-coupled NER (TC-NER), damage in transcribed strands of actively transcribed genes is recognized by stalled RNA polymerase molecules. In global genome NER (GG-NER), damage in the rest of the genome is initially recognized by the XPC and XPE proteins. These two proteins participate only in GG-NER, whereas the other XP proteins are required in both sub-pathways, as these converge after the recognition step (reviewed in Jeppesen *et al*.^4^). Following damage recognition, the damaged area is opened out by the multiprotein complex TFIIH, two of the subunits of which are the XPB and XPD proteins. XPA is involved in verification and correct placement of the proteins relative to the damage. XPF and XPG then cleave the DNA on either side of the damage, after which the resulting gap is filled in by DNA polymerases and ligases. NER is unaffected in XPV, which is caused by a deficit in translesion synthesis (TLS), the process involved in DNA replication past unrepaired damage in the template strand (reviewed in Lehmann^5^).

XP is a rare disease^1,6-8^ in which patients experience a variable combination of cutaneous, ophthalmological and neurological symptoms, as well as an increased risk of cancer.^1-3^ The cardinal cutaneous symptoms are exaggerated sunburn on minimal sun exposure in ∼50% of patients (especially in XPA, XPB, XPD, XPF and XPG),^7,9^ lentigines in sun-exposed areas,^1,10^ and markedly increased and earlier occurrence of skin cancers compared to the general population [including basal cell carcinoma (BCC), squamous cell carcinoma (SCC), and melanomas].^1,7,11,12^ Patients can develop conjunctival symptoms, corneal lesions and eyelid pathology.^1,2,13^ XP patients present with a 10- to 34-fold increased risk of extracutaneous cancer,^1,14^ especially for those affecting the CNS,^2,7,15^ the haematological system, the thyroid and gynaecological organs.^14^

In some series, XP neurological disease has been reported in 18–24% of patients.^1,7^ This phenotype has been well described in the Japanese XPA cohort,^16^ which constitutes a homogeneous group with an aggressive course, and other populations with severe XPA cases.^17,18^ In addition, XP neurological disease has been identified in XPB, XPD, XPF and XPG patients,^1,2,7^ including from mild forms to severe forms,^2^ and with childhood or adult onset.^19^ Slowly progressive features appear in most affected patients, and these can include cerebellar ataxia, hearing loss, learning disabilities or cognitive impairment, peripheral neuropathy, spasticity, chorea, dystonia and seizures.^1,17,19-29^ However, it is not known whether the complementation groups present with differential patterns of neurological symptoms and signs, and how the neurological disease progresses in the different groups over time. Although XPC, XPE and XPV have been classically considered not to display neurological features, an asymptomatic XP neurological disease (i.e. subclinical evidence of neurodegeneration) has been suggested in some XPC patients.^17,30^ This disease aspect has not been systematically investigated in previous studies, and it is unknown whether it could also affect XPE and XPV.

Several reports have described abnormalities in ancillary tests in patients with XP. Pure tone audiometry (PTA) can show sensorineural hearing loss,^24^ and electromyogram/nerve conduction studies (EMG/NCS) demonstrate a length-dependent sensorimotor or sensory neuropathy in some patients.^28,29^ Neuroimaging studies usually display cerebral and cerebellar atrophy,^31,32^ white matter lesions (preferentially in posterior regions), and bone abnormalities in some cases.^17,31,33^

XP presents with intergroup and intragroup clinical heterogeneity. The clinical variability between XP groups can be partially explained by the defective sub-pathways in each genotype. The TC-NER sub-pathway is preserved in XPC and XPE, and both sub-pathways are functional in XPV. Patients in these three groups usually present with normal sunburn reactions, lentigines in exposed skin areas, and a higher and earlier number of cutaneous cancers compared to the other groups.^2,7,9^ As mentioned, neurological involvement in these groups has been thought to be absent or minimal. In contrast, both GG-NER and TC-NER are defective in XPA, XPB, XPD, XPF and XPG. These patients experience exaggerated sunburn reactions and they have photoprotection implemented earlier than the other groups. This results in milder pigmentary changes, and lower occurrence of skin cancers. Patients in these groups may also present with XP neurological disease.^1,2^

Strict photoprotection, as well as surveillance and early treatment of suspicious lesions, are effective treatments for the UVR-related cutaneous and ophthalmological disease. Currently, no therapies can halt the progression of the XP neurological disease and, therefore, management remains symptomatic.^34,35^ Clinical development of novel therapeutics will require trials using valid outcome measures of disease progression. Although some attempts have been made to develop rating scales for the condition,^11^ the progression of the XP neurological disease has not been delineated using validated rating tools yet.

In this study, we aim to characterize the neurological disease in the UK XP cohort. We have investigated the neurological features in all the complementation groups, as well as their progression with validated rating tools. We will also present findings in patients’ ancillary tests and suggest some genotype-phenotype associations based on mutation severity.

## Materials and methods

### Study design and participants

We carried out a prospective cohort study based on a single tertiary centre that covers the entire UK population (UK National XP Service, St Thomas’ Hospital, Guys’ and St Thomas’ NHS Foundation Trust, London, UK). In the UK, the two diagnostic tests for XP (DNA repair assay and genetic analysis by a NER gene panel) are performed only in the two centres linked to the UK National XP Service (the Genome Damage and Stability Centre at the University of Sussex, and the Genetics Laboratory at Guy’s Hospital, respectively). Therefore, all newly diagnosed cases are communicated to the UK National XP Service, and patients are offered clinical care by the medical team. Participants’ consent was obtained according to the Declaration of Helsinki, and the study was approved by the Research Ethics Committee of Guy’s and St Thomas’ NHS Foundation Trust (reference number: 12/LO/0325). Collected data span the period from December 2009 to August 2021.

Paediatric and adult participants with a clinical diagnosis of XP (with confirmation of abnormal DNA repair activity)^36^ were recruited to the study. XP groups were assigned through complementation assay^37^ and genetic testing. Participants received a multidisciplinary assessment, and each specialist recorded clinical data in standardized paper forms specific to each specialty (dermatology, ophthalmology, neurology, neuropsychology and clinical genetics). Medical records were reviewed to collect retrospective data on events preceding baseline visits. These data were confirmed with participants and/or relatives/carers, if possible. Participants were offered ancillary tests and these were performed following clinical protocols. Follow-up visits were carried out during participants’ clinical visits. These are normally organized on an annual basis, although this could vary for clinical reasons.

Collected data included demographics, genetic information, comorbidities, medication, developmental history, age of onset for several events in the condition and first manifestation for each affected system (skin, eye, nervous system). Since cerebellar ataxia was the most frequent motor syndrome previously reported in the literature,^1,16,17^ and in preliminary observations in our cohort, the adult neurologist for the UK National XP Service (P.G.) selected three different tools validated in other ataxic conditions to assess participants’ neurological status and examination. The Scale for the Assessment and Rating of Ataxia (SARA) is a clinical scale for the standardized examination and severity scoring of cerebellar signs in patients with cerebellar ataxia.^38^ The Inventory of Non-Ataxia Signs (INAS) is a clinical tool used to record the presence of extracerebellar findings.^39^ It considers reflexes, motor signs, vibration sense impairment, neuro-ophthalmological findings and reported abnormalities. The INAS total count results from the presence (1 point) or absence (0 points) of 16 of the signs. The Activities of Daily Living questionnaire (ADL) is a subscale included in the Friedreich’s Ataxia Rating Scale (FARS), aimed at assessing participants’ performance in basic daily activities.^40^

### Diagnostic tests

To assess unscheduled DNA synthesis (UDS), direct scintillation counting of ^3^H-thymidine incorporation into the repaired DNA was measured, modified from the procedure previously described.^36^ XPV cell lines were identified by having normal UDS but sensitization by caffeine to the lethal effects of UVR.^41^ Complementation analysis allows the classification of patients in different XP groups through fusion of a patient’s fibroblasts with fibroblasts from donors of known XP groups, as previously reported.^37^ More recently, complementation group and causative mutations were identified through molecular analysis.^2^

To study genotype-phenotype associations, one of the authors with expertise in the molecular genetic analysis of XP patients (A.R.L.) created a score to rate the severity of the mutations (Supplementary Table 1), with higher ranks representing more severe deficits in protein function. This score is based on *in silico* predicted mutation effects and refined through results from UDS and other functional assays. The score was further discussed with two authors with clinical expertise (H.G.M. and P.G.).

### Ancillary tests

Brain and spine MRI, EMG/NCS and PTA were performed following standard clinical protocols. MRI were performed in different 1.5 T and 3 T clinical scanners, and sequences included T_1_, T_2_, FLAIR, echo T_2_* and diffusion weighted imaging (DWI). Qualitative variables were extracted from the reports of these tests.

### Statistical analysis

Statistical analyses were performed in R (version 4.1.2) and Stata (version 17.0).

#### Survival analysis

Age of onset for different events in the condition was analysed with the Kaplan-Meier method. Kaplan-Meier estimates of median survival times (p50) and their 95% confident intervals (CI) were reported for each event in the different complementation groups. Occasionally, there were insufficient events to compute these estimates (noted as N/A in Table 1). Kaplan-Meier curves and global log-rank test results are presented. Dot plots containing the age of onset for the subjects who presented the event of interest are also included for data visualization purposes.

**Table 1:** Characterization of the study cohort

	XPA	XPB	XPC	XPD	XPE	XPF	XPG	XPV
Number of subjects [*n* (% relative to total sample)]	21 (22.6)	2 (2.2)	22 (23.7)	17 (18.3)	7 (7.5)	4 (4.3)	8 (8.6)	12 (12.9)
Number of visits per patient [median (Q1, Q3)]	5.0 (4.0, 7.0)	3.5 (2.3, 4.8)	4.0 (3.0, 6.0)	5.0 (4.0, 7.0)	2.0 (2.0, 4.0)	3.0 (2.0, 4.5)	4.5 (3.0, 6.3)	4.0 (2.8, 5.3)
Age at baseline [years, median (Q1, Q3)]	24.5 (13.2, 34.5)	42.9 (37.8, 47.9)	11.9 (8.6, 22.5)	22.8 (13.8, 32.8)	44.7 (35.8, 52.2)	23.3 (20.2, 29.0)	17.3 (13.9, 37.3)	57.0 (46.0, 64.5)
Follow-up time [years, mean (95% CI)]^a^	3.8 (2.8, 4.7)	N/A	3.8 (2.8, 4.7)	4.1 (3.0, 5.2)	2.4 (0.7, 4.1)	N/A	2.5 (1.0, 4.1)	3.7 (2.4, 5.0)
UDS [%, median (Q1, Q3)]^b^	5.0 (2.0, 12.0)	10.0 (10.0, 10.0)	12.5 (9.0, 15.0)	23.5 (16.0, 25.0)	60.5 (53.0, 68.0)	25.0 (12.5, 35.0)	2.0 (1.0, 3.0)	99.0 (91.0, 111.0)
Age of disease onset [years, median (95% CI)]^c^	2.5 (2.5, 4.5)	0.5 (N/A, N/A)	2.5 (2.5, 3.5)	0.5 (0.5, 0.5)	12.5 (3.5, N/A)	2.0 (0.5, N/A)	0.5 (0.5, N/A)	18.0 (7.5, N/A)
Age of diagnosis [years, median (95% CI)]^c^	13.5 (6.5, 34.5)	36.5 (28.5, N/A)	4.5 (3.5, 6.5)	3.5 (3.5, 10.5)	41.5 (29.5, N/A)	9.0 (6.5, N/A)	8.5 (3.5, N/A)	45.5 (24.5, N/A)
Age of skin symptoms onset [years, median (95% CI)]^c^	2.5 (2.5, 4.5)	0.5 (N/A, N/A)	2.5 (2.5, 3.5)	0.5 (0.5, 0.5)	12.5 (3.5, N/A)	2.0 (0.5, N/A)	0.5 (0.5, N/A)	18.0 (7.5, N/A)
Age of eye symptoms onset [years, median (95% CI)]^c^	15.5 (7.5, N/A)	32.7 (12.5, N/A)	7.5 (4.5, N/A)	12.5 (3.5, N/A)	49.5 (49.5, N/A)	N/A (N/A, N/A)	16.5 (5.5, N/A)	59.5 (44.5, N/A)
Age of neurological symptoms onset [years, median (95% CI)]^c^	16.5 (8.5, N/A)	27.6 (17.5, N/A)	N/A (N/A, N/A)	12.5 (8.5, N/A)	N/A (N/A, N/A)	N/A (11.5, N/A)	28.5 (4.5, N/A)	N/A (N/A, N/A)
Age of cutaneous cancer onset [years, median (95% CI)]^c^	59.5 (59.5, N/A)	30.1 (22.5, N/A)	20.5 (16.5, N/A)	22.5 (13.5, N/A)	22.5 (14.5, N/A)	N/A (N/A, N/A)	N/A (N/A, N/A)	38.5 (28.5, N/A)
Neurological symptoms [*n* (% relative to group size)]	12 (57.1)	1 (50.0)	2 (9.1)	13 (76.5)	0 (0.0)	1 (25.0)	7 (87.5)	0 (0.0)

N/A = not applicable; Q1 = first quartile; Q3 = third quartile; UDS = unscheduled DNA synthesis; 95% CI = 95% confidence interval.

^a^ *P*-values for contrasts between groups, corrected via the Tukey’s method, were all *P* > 0.526.

^b^Statistically significant differences among groups, *P* < 0.001 in Kruskal-Wallis test.

^c^If there were insufficient events to calculate median survival times and/or their 95% CIs, N/A is indicated.

#### Cross-sectional analysis of rating tools

Repeated measures ANOVA with random participant effects were used for cross-sectional analyses of SARA and ADL items and INAS total count. Degrees of freedom were calculated with the Kenward-Roger method. Estimated mean scores and their 95% CI are presented for each item and group. Global Chi-squared (χ^2^) tests are presented for each item and, if statistically significant (*P* < 0.05), pairwise contrasts were calculated and corrected for multiple comparisons via the Tukey’s method. Additional models were fitted including age as a covariable, using a flexible restricted cubic spline function of age with five degrees of freedom.

We used Bayesian logistic regression models with random participant effects and nearly uninformative prior probabilities to analyse INAS item frequencies among complementation groups. This approach was chosen as frequentist methods are unstable due to rare frequencies of positive INAS signs within some groups. Estimated mean frequencies are presented. Pairwise contrasts were calculated, and their significance assessed through 95% credible intervals.

#### Longitudinal analysis of rating tools

Linear mixed models with random slopes and random intercepts were used for the longitudinal analysis of SARA and ADL items and INAS total count (R, version 4.1.2; lme4 library, version 1.1–27.1). Different models were computed to estimate progression rates as a function of follow-up time (both unadjusted and adjusted by baseline age), time since onset of any symptom of the condition, and time since onset of the neurological disease. Estimated progression rates (points per year) and their 95% CI are presented for each item and group. Global χ^2^ tests are presented for each item, and, if statistically significant (*P* < 0.05), pairwise contrasts were calculated and corrected for multiple comparisons via the Tukey’s method.

#### Lasso method for model selection

Multinomial logistic regression models were fitted using a least absolute shrinkage and selection operator (Lasso) method for model selection, in order to explore whether combinations of SARA, ADL or INAS items could predict a patient’s complementation group (see Supplementary material, Text 1 and Supplementary Fig. 1 for a brief description of the Lasso method). We used ‘grouped’ retention of predictor variables (i.e. we forced the same predictor variables to be used for all group comparisons). Final models were determined by 5-fold cross-validation using the ‘one standard error’ (1-SE) rule to choose the final Lasso penalty.^42^ We used the R glmnet package (version 1.0).^43^

#### Effects of mutation severity

Linear mixed models with random slopes were used to investigate the effects of the mutation severity score on SARA and ADL total scores, and their rates of progression (unadjusted and adjusted by complementation group). Since mutation severity will affect the groups differently, effects of mutation severity score were investigated in individual groups. Only XPA, XPD, XPG and XPV groups showed enough variation in their mutation severity scores to perform the analyses.

Because of small within-complementation group sample sizes, we assessed within-group associations of mutation severity and age of onset for different events using log-rank tests for ordinal association with censored data. *P*-values testing against marginal null distributions were approximated via 10 000 random permutations of the observed data using the *coin* package (version 1.4–2) within R. We report the estimated log-rank coefficient (lrank) and null-permutation *P*-value estimate.

## Results

### Cohort description

Our study comprised 93 patients with XP, including information from 417 visits (4.48 visits/patient, on average) (Table 1). XPB and XPF were extremely rare in our sample (*n* = 2 and *n* = 4, respectively), and these groups were considered only for descriptive purposes. Follow-up time was not statistically different among groups.

### Age of disease onset and age of diagnosis

**Figure 1:**
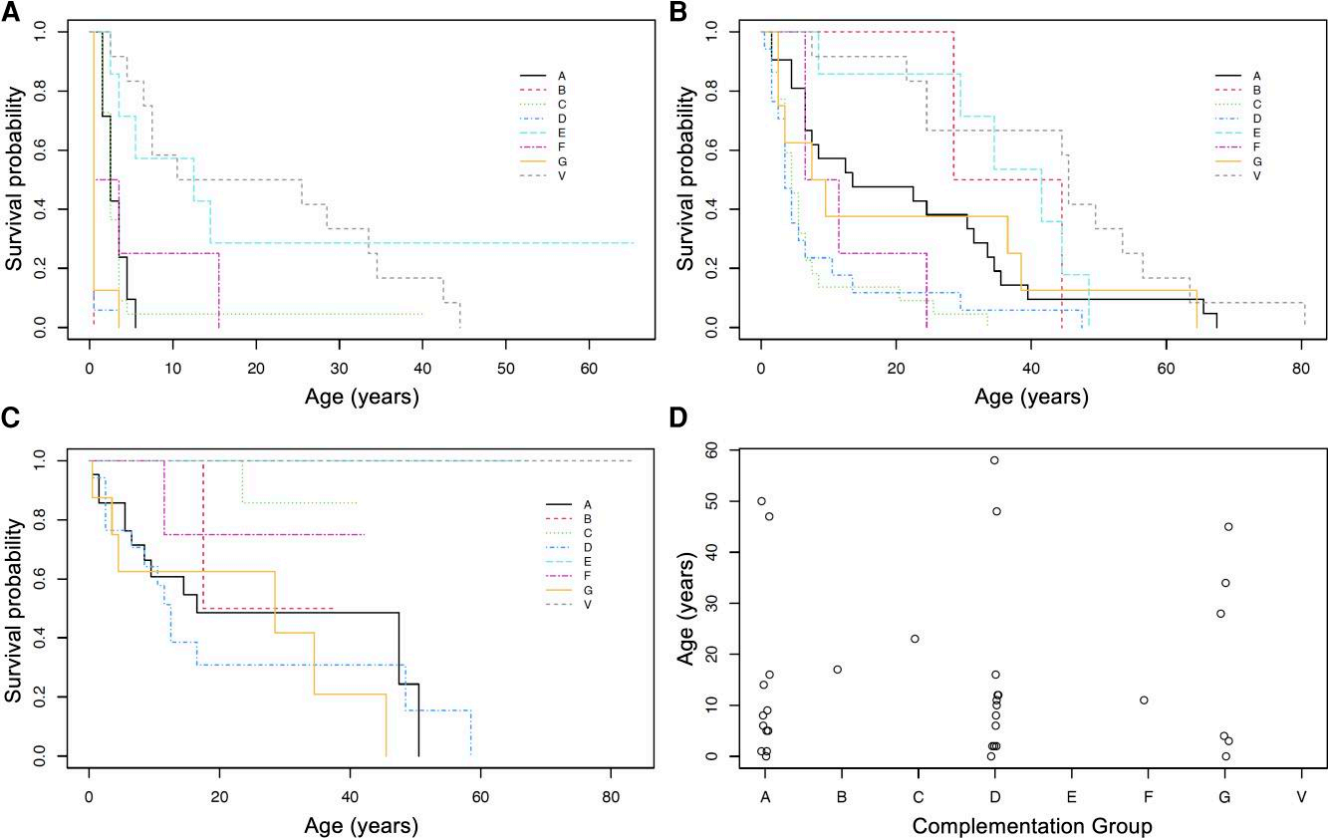
**Age of occurrence for different events in patients with xeroderma pigmentosum.** (**A**) Kaplan-Meier plot for age of onset of the first feature of the condition in the different complementation groups (global log-rank test, χ^2^ = 80.3, df = 5, *P* < 0.001). (**B**) Kaplan-Meier plot for age of diagnosis of the condition in the different complementation groups (global log-rank test, χ^2^ = 38.9, df = 5, *P* < 0.001). (**C**) Kaplan-Meier plot for age of onset of neurological symptoms in the different complementation groups (global log-rank test, χ^2^ = 46.1, df = 5, *P* < 0.001). (**D**) Dot plot representing individual values for age of onset of neurological symptoms in those subjects who presented the event of interest.

Median survival times for different events in the XP groups are summarized in Table 1. XPD and XPG showed a very early onset for the condition (with at least 50% of patients showing symptoms before the first year of age) (Fig. 1A). Other groups showed a later age of onset, especially XPE and XPV. The first symptoms displayed by patients were mainly severe or exaggerated sunburn (in XPA, XPB, XPD, XPF and XPG) or lentigines (in XPC, XPE and XPV) (Supplementary Table 2). Regarding the diagnosis of the condition (Fig. 1B), many XPC and XPD cases were diagnosed at an early age, having short median diagnostic delays (2 and 3 years, respectively) (Supplementary Table 3). On the contrary, XPV and XPE were diagnosed later in life and showed longer diagnostic delays (27.5 and 29 years, respectively).

### Occurrence and onset of neurological symptoms in patients with xeroderma pigmentosum

Overall, 36 patients (38.7% of the total cohort) presented with neurological symptoms (Table 1). More than 50% of patients in the XPG, XPD and XPA groups had neurological symptoms, whereas these were absent in XPE and XPV patients. The most common initial neurological symptoms were imbalance (8.6% of the total sample), neurodevelopmental delay (7.5%), cognitive symptoms (7.5%) and hearing impairment (7.5%) (Supplementary Table 4). Kaplan-Meier and dot plots suggest the existence of two subgroups in XPA, XPD and XPG (Fig. 1C and D). In XPA and XPD, the first group would have an onset below 20 years of age, and the second group, an onset above 45 years of age. For XPG, there is a gap between 4 and 28 years of age in which patients do not present an onset for neurological symptoms.

### Age of onset for other disease manifestations and insights into the natural history of xeroderma pigmentosum

As a whole, median survival times for the different symptoms could suggest a sequence of events in some XP patients (Table 1). Skin symptoms are the earliest phenomena (Supplementary Fig. 2 and Supplementary Table 5), marking the onset of the disease and leading to diagnosis in most cases (unless those symptoms are very subtle). Second, ophthalmological symptoms and signs are recognized (Supplementary Fig. 3 and Supplementary Table 6), with neurological features subsequently appearing. Cutaneous cancers would emerge later in life, as these tend to be the result of accumulated actinic damage (Supplementary Fig. 4 and Supplementary Table 7).

### Cause of death in xeroderma pigmentosum and neuropathological data

Eight patients passed away during the study: two XPA participants (aged 36, from pneumonia, and 84 years), two XPB cases (one of them aged 55, affected by lung cancer), two XPD patients (aged 28 and 51 years, from sepsis and pneumonia, respectively) and two XPG (aged 9 and 69 years, from urinary sepsis and pulmonary embolism, respectively). Description of the neuropathological findings in the XPD patient who passed away at the age of 28 was available (Supplementary material, Text 2 and Supplementary Figs 5 and 6).

### Group profiles with clinical rating tools and annual progression rates

#### Scale for the Assessment and Rating of Ataxia

Estimated SARA total mean scores and their CIs were above the threshold for non-ataxic subjects (≥3 points) in XPD [11.24 (95% CI: 7.16, 15.32)], XPG [10.99 (95% CI: 5.22, 16.77)] and XPA [7.32 (95% CI: 3.68, 10.97)]. Conversely, XPV, XPE and XPC showed estimated mean scores <3 points, and their CIs included the null value (Table 2). SARA items showed a similar pattern, with mean scores >0 in XPA, XPD and XPG, but not in XPC, XPE and XPV (except in XPV for SARA nose-finger, which indicates the presence of intention tremor) (Table 2).

**Table 2:** Cross-sectional analysis of SARA items with repeated measures ANOVA with random groups effects^a^

SARA item	XPA	XPC	XPD	XPE	XPG	XPV	Global χ^2^ test	Statistically significant pairwise contrasts
SARA gait	1.72 (0.98, 2.47)	0.10 (−0.63, 0.83)	2.36 (1.54, 3.19)	0.24 (−1.06, 1.54)	2.48 (1.27, 3.68)	0.38 (−0.60, 1.37)	25.95, df = 5, *P* < 0.001	G versus C D versus C, V A versus C
SARA stance	1.25 (0.70, 1.79)	0.04 (−0.49, 0.57)	1.60 (0.97, 2.22)	0.07 (−0.88, 1.03)	1.69 (0.80, 2.57)	0.16 (−0.57, 0.88)	24.84, df = 5, *P* < 0.001	G versus C D versus C, V A versus C
SARA sitting	0.40 (0.07, 0.72)	0.00 (−0.32, 0.32)	0.65 (0.28, 1.03)	0.00 (−0.58, 0.58)	0.56 (0.03, 1.10)	0.00 (−0.44, 0.44)	11.29, df = 5, *P* = 0.046	Non-significant (D versus C, *P* = 0.099)
SARA speech	1.28 (0.63, 1.92)	0.00 (−0.63, 0.63)	2.17 (1.46, 2.89)	0.00 (−1.13, 1.13)	1.35 (0.31, 2.40)	0.11 (−0.74, 0.97)	26.26, df = 5, *P* < 0.001	D versus C, E, V
SARA finger chase	0.55 (0.20, 0.90)	0.04 (−0.30, 0.37)	0.80 (0.42, 1.18)	0.00 (−0.60, 0.60)	0.91 (0.35, 1.47)	0.19 (−0.27, 0.64)	15.32, df = 5, *P* = 0.009	D versus C
SARA nose-finger	0.54 (0.21, 0.87)	0.15 (−0.17, 0.46)	0.78 (0.42, 1.14)	0.22 (−0.35, 0.78)	0.96 (0.44, 1.49)	0.57 (0.14, 0.99)	11.99, df = 5, *P* = 0.035	Non-significant (D versus C, *P* = 0.094; G versus C, *P* = 0.092)
SARA fast alternating hand movements	1.03 (0.55, 1.50)	0.13 (−0.32, 0.59)	1.38 (0.85, 1.91)	0.14 (−0.67, 0.95)	1.38 (0.62, 2.13)	0.37 (−0.25, 0.98)	19.90, df = 5, *P* = 0.001	D versus C
SARA heel-shin	0.94 (0.51, 1.38)	0.00 (−0.41, 0.42)	1.25 (0.78, 1.72)	0.04 (−0.72, 0.78)	1.45 (0.76, 2.14)	0.01 (−0.56, 0.58)	28.22, df = 5, *P* < 0.001	G versus C, V D versus C, V A versus C
SARA total	7.32 (3.68, 10.97)	0.47 (−3.01, 3.95)	11.24 (7.16, 15.32)	0.69 (−5.51, 6.89)	10.99 (5.22, 16.77)	1.79 (−2.93, 6.50)	23.77, df = 5, *P* < 0.001	D versus C, V G versus C

^a^Results represent estimated mean scores in each complementation group (accounting for intrasubject variability), with their 95% CIs. χ^2^ values represent the test for global differences among the groups. If such test is significant (*P* < 0.05), pairwise contrasts are calculated and corrected for multiple comparisons via the Tukey’s method. Statistically significant contrasts for each item (*P* < 0.05) are summarized in the last column.

There were statistically significant global differences among the groups for all items and the total score. Pairwise comparisons showed similar patterns in most items, with XPA, XPD or XPG being different from XPC, XPE and/or XPV. However, no differences among XPA, XPD and XPG (or among XPC, XPE and XPV) were found. Age did not produce clinically significant changes in the estimated mean scores.

Estimated SARA total annual progression rates as a function of follow-up time were statistically significant in XPD [0.91 points per year (95% CI: 0.61, 1.21)] and XPA [0.63 points per year (95% CI: 0.38, 0.89)]. Although XPG showed some progression in SARA total score, this did not reach the level of significance (Table 3). There were overall differences among the groups, and pairwise comparisons were significant in XPD versus XPC and XPV, as well as XPA versus XPC. Estimated annual progression rates were also calculated as a function of time since onset of the first symptom of the condition, and time since onset of neurological symptoms. These estimated rates were similar to the ones estimated as a function of follow-up time, especially for XPA and XPD (Supplementary Table 8).

**Table 3:** Longitudinal analysis of rating tools data^a^

Rating tool	XPA	XPC	XPD	XPE	XPG	XPV	Global χ^2^ test	Statistically significant pairwise contrasts
SARA total	0.63 (0.38, 0.89)	−0.03 (−0.26, 0.19)	0.91 (0.61, 1.21)	0.05 (−0.52, 0.62)	0.54 (−0.07, 1.15)	0.07 (−0.29, 0.43)	32.37, df = 5, *P* < 0.001	D versus C, V A versus C
ADL total	0.42 (0.16, 0.67)	−0.01 (−0.22, 0.21)	0.55 (0.19, 0.92)	0.07 (−0.51, 0.65)	0.36 (−0.32, 1.04)	0.16 (−0.28, 0.60)	10.39, df = 5, *P* = 0.065	Non-significant (D versus C, *P* = 0.097; A versus C, *P* = 0.131)
INAS total	0.19 (0.04, 0.34)	−0.02 (−0.14, 0.11)	0.36 (0.19, 0.52)	0.14 (−0.21, 0.49)	−0.18 (−0.51, 0.14)	0.03 (−0.20, 0.26)	17.66, df = 5, *P* = 0.003	D versus C, G

ADL = Activities of Daily Living questionnaire; INAS = Inventory of Non-Ataxia Signs; SARA = Scale for the Assessment and Rating of Ataxia.

^a^Results represent estimated mean progression rates (points per year), as a function of follow-up time, for each total score in each complementation group (accounting for intrasubject variability), with their 95% CIs. χ^2^ values represent the test for global differences among progression rates in the groups. If such test is significant (*P* < 0.05), pairwise contrasts are calculated and corrected for multiple comparisons via the Tukey’s method. Statistically significant contrasts for each total score (*P* < 0.05) are summarized in the last column.

Annual progression rates as a function of follow-up time for all SARA items are summarized in Supplementary Table 9. There were statistically significant differences among groups in SARA gait, SARA stance and SARA speech progression rates.

#### Activities of Daily Living Questionnaire

Estimated ADL total mean scores indicated a mild-to-moderate level of disability in XPD [10.26 (95% CI: 6.13, 14.38)], XPG [7.87 (95% CI: 2.02, 13.72)] and XPA [7.08 (95% CI: 3.47, 10.69)]. Estimated ADL total mean scores in XPV, XPE and XPC pointed towards absence of neurological disability in these groups (Supplementary Table 10). ADL speech and ADL walking were among the most affected items in XPA, XPD and XPG (Supplementary Table 10). Pairwise comparisons showed statistically significant differences in some ADL items between XPA, XPD or XPG and the other three groups, but not among XPA, XPD and XPG (Supplementary Table 10). Age did not show statistically significant effects on the individual ADL items or the total score.

Estimated ADL total progression rates as a function of follow-up time were statistically significant in XPD [0.55 points per year (95% CI: 0.19, 0.92)] and XPA [0.42 points per year (95% CI: 0.16, 0.67)] (Table 3). However, global differences in progression rates among the groups could not be demonstrated (χ^2^ = 10.39, df = 5, *P* = 0.065). ADL speech, ADL dressing and ADL walking progression rates were significantly different in XPD when compared to XPC (Supplementary Table 11).

#### Inventory of Non-Ataxia Signs

Limb hyporeflexia and hypopallesthaesia were frequent extracerebellar features in patients, especially in XPD, XPA and XPG (Table 4 and Supplementary Table 12). These were also frequent in XPV cases, despite the absence of reported neurological symptoms in this group.

**Table 4:** Estimated mean frequencies of INAS items in the different complementation groups^a^

INAS item	XPA	XPC	XPD	XPE	XPG	XPV	Statistically significant contrasts (95% credible intervals)
UL hyporeflexia	54.3	5.7	74.2	15.4	30.2	23.4	D versus C, E, G, V A versus C, E, V G versus C
LL hyporeflexia	47.5	3.4	85.8	12.4	34.3	53.2	D versus A, C, E, G, V V versus C, E A versus C G versus C
Hypopallesthaesia	16.7	3.5	37.1	12.1	7.5	17.4	D versus C, G A versus C
UL hyperreflexia	2.0	9.6	0.2	0.0	22.7	0.0	G versus A, D, E, V C versus D, V
LL hyperreflexia	3.1	14.1	11.3	0.0	41.4	0.2	G versus A, D, E, V
Babinski sign	10.6	0.6	25.8	0.0	56.2	0.1	G versus A, C, E, V D versus C, E, V A versus C
LL spasticity	20.7	7.4	28.3	0.0	30.7	0.1	G versus C, E, V D versus C, E, V A versus E, V
Distal UL paresis	9.9	0.7	13.8	0.0	27.6	20.3	G versus C, E V versus C, E D versus C A versus C
Distal LL paresis	12.8	0.3	24.8	0.0	29.8	3.5	G versus C, E, V D versus C, E, V A versus C, E
Bulbar atrophy	4.0	0.5	2.6	0.0	21.2	3.8	G versus C, E
Distal UL atrophy	7.0	0.8	9.1	0.0	17.1	6.8	G versus C
Distal LL atrophy	11.8	0.5	20.1	0.0	28.5	6.7	G versus C, E D versus C, E A versus C, E
Craniocervical chorea	3.2	0.4	11.2	0.0	32.8	0.0	G versus A, C, E, V D versus C, E, V
UL chorea	5.8	0.4	23.1	0.0	34.7	0.0	G versus A, C, E, V D versus A, C, E, V
Craniocervical dystonia	9.5	0.3	7.0	0.0	31.4	0.0	G versus A, C, D, E, V A versus C, E, V D versus C
UL dystonia	13.3	8.7	22.7	5.4	20.9	0.0	G versus V D versus V A versus V
Axial myoclonus	1.2	0.2	0.1	18.3	6.2	2.6	E versus A, C, D
Altered ocular pursuit	34.3	1.7	49.8	0.0	46.0	18.4	D versus C, E, V G versus C, E A versus C, E, V versus C, E
Hypometric saccades	25.5	7.9	40.8	0.0	48.8	16.4	G versus C, E D versus C, E A versus C, E V versus E
Horizontal ophthalmoparesis	9.3	1.0	23.0	0.0	0.1	0.1	D versus C, E, G, V
Vertical ophthalmoparesis	27.8	1.8	31.9	0.0	32.2	7.7	G versus C, E D versus C, E A versus C, E
Slowness of saccades	21.2	4.1	36.9	0.0	26.7	0.0	D versus C, E, V G versus C, E, V A versus C, E, V
Urinary dysfunction	17.2	8.5	27.2	0.0	38.1	12.7	G versus C, E D versus E
Cognitive impairment	58.4	17.6	85.0	15.1	83.2	4.3	D versus C, E, V G versus C, E, V A versus C, E, V

INAS = Inventory of Non-Ataxia Signs; LL = lower limb; UL = upper limb.

^a^Data for all the INAS items can be found in Supplementary Table 12. Frequencies are presented as percentages. Frequencies were calculated using Bayesian methods to account for intrasubject variability.

Hyperreflexia, Babinski sign, spasticity and muscle atrophy were especially frequent in XPG. Spasticity was also present in XPD and XPA cases. Weakness was preferentially distal in lower limbs in XPD, XPG and XPA. Three XPV cases presented with distal upper limb weakness.

Chorea and dystonia were the most frequent types of movement disorders in XP (Table 4 and Supplementary Table 12). Chorea was present in XPG and XPD (in craniocervical regions and upper limbs), and in one subject with XPB (multiple regions, data not shown).^27^ Dystonia was frequent in XPA and XPD (in upper limbs), and in XPG (in craniocervical regions and upper limbs). Interestingly, some XPC patients displayed upper limb dystonia when performing Fogs’ feet-hands test. Axial myoclonus was found in two XPE subjects.

Impaired smooth pursuit, hypometric saccades, slowness of saccades (especially in initiation) and vertical ophthalmoparesis (particularly in downgaze) were common oculomotor findings in XPD, XPG and XPA (Table 4 and Supplementary Table 12). The range of horizontal ocular movements was preserved more frequently than in the vertical plane for all the groups.

Cognitive impairment was a frequent feature in XPD, XPG and XPA. This was even reported in some XPC and XPE cases (Table 4 and Supplementary Table 12).

Estimated INAS total mean counts in XPG [4.28 (95% CI: 2.99, 5.57)], XPD [3.73 (95% CI: 2.82, 4.63)] and XPA [2.53 (95% CI: 1.73, 3.32)] were significantly different from the scores in XPV, XPC and/or XPE. Estimated INAS total annual progression rates were statistically significant in XPD and XPA (Table 3), indicating some measurable progression in the complexity of patients’ phenotypes in these groups.

#### Group prediction using extracerebellar features: Lasso model selection for INAS items

We investigated whether a subset of INAS items accurately predicts a patient’s complementation group. By excluding the oculomotor signs and the reported abnormalities (both frequently missing), the number of patients’ visits used to fit the model increased from 53.8% to 68.4%.

The 1-SE cross-validated Lasso model selected only eight predictors (Table 5). These predictors seemed to follow clearly distinguished patterns. For instance, XPA would be characterized by upper limb hyporeflexia and gait spasticity. XPD would show upper and lower limb hyporeflexia as main features. Lower limb hyperreflexia and Babinski sign would be more typical of XPG. XPC and XPE would typically lack any of the signs in the model. Presence of lower limb hyporeflexia and distal upper limb paresis, with absence of the other signs, would point towards XPV.

**Table 5:** Multinomial logistic regression coefficients of the one standard error (1-SE) cross-validated Lasso model for a reduced set of INAS items^a^

INAS item	XPA	XPC	XPD	XPE	XPG	XPV
LL hyperreflexia	**−0**.**74**	0.11	**0**.**42**	−0.38	**1**.**17**	**−0**.**58**
UL hyporeflexia	**1**.**11**	**−0**.**52**	**0**.**65**	−0.25	0.22	**−1**.**21**
LL hyporeflexia	−0.14	**−1**.**94**	**1**.**76**	**−0**.**55**	−0.33	**1**.**21**
Babinski sign	−0.34	**−0**.**78**	0.28	−0.24	**1**.**43**	−0.35
Gait spasticity	**0**.**46**	0.00	−0.14	−0.08	−0.07	−0.17
LL spasticity	0.14	0.02	0.00	−0.04	−0.05	−0.07
Distal UL paresis	0.01	−0.32	−0.29	−0.10	0.28	**0**.**43**
LL rigidity	0.14	−0.04	−0.08	−0.01	0.01	−0.02

INAS = Inventory of Non-Ataxia Signs; LL = lower limb; UL = upper limb.

^a^Coefficients are presented as log(odds). A positive coefficient (>0) indicates that the presence of an item increases the odds of being classified in a complementation group. A negative coefficient (<0) indicates that the presence of an item decreases the odds of being classified in a complementation group. A coefficient close to the null value represents that the frequency of a sign in a complementation group is close to the average frequency of the other groups. Coefficients above the |±0.4| threshold are in bold.

The accuracy of the model was 52.3% when patients were assigned to the complementation group with highest estimated probability. If the relative baseline frequencies of the groups are ignored and classification is based on the highest odds ratio, the model accuracy was 45.4%. The classification frequencies with the latter rule are presented in Supplementary Table 13. XPC, XPD and XPG were correctly classified in a high number of cases. XPA was misclassified as XPC and XPD, whereas XPE and XPV were misclassified as XPC.

#### Group prediction using cerebellar features and disability variables: Lasso model selection for SARA and ADL items

To study whether any patterns in SARA and ADL could predict a patient’s complementation group, the items of both scales were combined to fit multinomial logistic regression models, using the Lasso method. For these models, 69.7% of the visits were used, as they had full SARA and ADL scores.

The 1-SE model (Supplementary Table 14) indicated that XPA and XPD are characterized by higher scores in SARA gait, SARA speech and ADL speech, whereas XPG is characterized only by higher scores in SARA gait. XPC and XPE would have low scores in most items, especially SARA gait and ADL speech. XPV would typically present with higher scores in SARA nose-finger, but lower scores in SARA gait and ADL speech. Classification performance resulted in an accuracy of 42.1% (highest probability rule) and 49.2% (maximum odds ratio rule). XPC showed the highest proportion of correctly classified visits.

### Ancillary tests in patients with xeroderma pigmentosum

The proportion of abnormal PTA was higher in XPB (100%), XPG (75.0%) and XPD (70.6%) (Table 6 and Supplementary Table 15). In most groups, sensorineural hearing loss (SNHL) was the most frequent type of impairment. In XPE and XPV, hearing loss was typically mild. However, in XPA, XPB, XPD and XPG, more than 50.0% of patients with abnormal audiograms presented with moderate-severe hearing loss.

**Table 6:** Characterization of ancillary tests in patients with xeroderma pigmentosum^a^

	XPA	XPB	XPC	XPD	XPE	XPF	XPG	XPV
**Pure tone audiometry**								
Subjects with abnormal audiogram [*n*, (% over total group size)]	6 (28.6)	2 (100)	1 (4.5)	12 (70.6)	2 (28.6)	0 (0.0)	6 (75.0)	4 (33.3)
Hearing loss type								
SNHL [*n* (%)]	6 (100)	2 (100)	0 (0.0)	11 (91.7)	2 (100)	N/A	4 (66.7)	3 (75.0)
Conductive [*n* (%)]	0 (0.0)	0 (0.0)	1 (100)	0 (0.0)	0 (0.0)	N/A	1 (16.7)	0 (0.0)
Mixed [*n* (%)]	0 (0.0)	0 (0.0)	0 (0.0)	1 (8.3)	0 (0.0)	N/A	0 (0.0)	0 (0.0)
Hearing loss severity								
Mild [*n* (%)]	2 (33.3)	0 (0.0)	0 (0.0)	4 (33.3)	2 (100)	N/A	1 (16.7)	3 (75.0)
Moderate [*n* (%)]	3 (50.0)	0 (0.0)	1 (100)	6 (50.0)	0 (0.0)	N/A	2 (33.3)	1 (25.0)
Severe [*n* (%)]	1 (16.7)	2 (100)	0 (0.0)	1 (8.3)	0 (0.0)	N/A	3 (50.0)	0 (0.0)
**EMG/NCS**								
Subjects with abnormal EMG/NCS [*n* (% over total group size)]	3 (14.3)	0 (0.0)	1 (4.5)	11 (64.7)	0 (0.0)	0 (0.0)	4 (50.0)	1 (8.3)
Type of neuropathy								
Axonal [*n* (%)]	2 (66.7)	N/A	0 (0.0)	7 (63.6)	N/A	N/A	3 (75.0)	1 (100)
Mixed [*n* (%)]	0 (0.0)	N/A	0 (0.0)	2 (18.2)	N/A	N/A	1 (25.0)	0 (0.0)
Type of affected fibres								
Sensory [*n* (%)]	2 (66.7)	N/A	1 (100)	8 (72.7)	N/A	N/A	2 (50.0)	0 (0.0)
Sensorimotor [*n* (%)]	1 (33.3)	N/A	0 (0.0)	3 (27.3)	N/A	N/A	2 (50.0)	1 (100)
Severity								
Mild [*n* (%)]	0 (0.0)	N/A	0 (0.0)	3 (27.3)	N/A	N/A	3 (75.0)	1 (100)
Moderate [*n* (%)]	2 (66.7)	N/A	0 (0.0)	4 (36.4)	N/A	N/A	0 (0.0)	0 (0.0)
Severe [*n* (%)]	1 (33.3)	N/A	0 (0.0)	1 (9.1)	N/A	N/A	1 (25.0)	0 (0.0)
**MRI**								
Subjects with abnormal MRI [*n* (% over total group size)]	6 (28.6)	2 (100)	6 (27.3)	12 (70.6)	1 (14.3)	1 (25.0)	7 (87.5)	2 (16.7)
Findings								
Isolated infratentorial atrophy [*n* (%)]	1 (16.7)	0 (0.0)	1 (16.7)	0 (0.0)	0 (0.0)	0 (0.0)	0 (0.0)	0 (0.0)
Isolated supratentorial atrophy [*n* (%)]	0 (0.0)	0 (0.0)	1 (16.7)	3 (25.0)	0 (0.0)	0 (0.0)	0 (0.0)	0 (0.0)
Global atrophy [*n* (%)]	5 (83.3)	2 (100)	1 (16.7)	8 (66.7)	0 (0.0)	1 (100)	3 (42.9)	1 (50.0)
Cerebellar atrophy [*n* (%)]	5 (83.3)	2 (100)	2 (33.3)	7 (58.3)	0 (0.0)	1 (100)	2 (28.6)	0 (0.0)
Focal lesion [*n* (%)]	2 (33.3)	0 (0.0)	2 (33.3)	1 (8.3)	0 (0.0)	0 (0.0)	1 (14.3)	2 (100)
White matter changes [*n* (%)]	2 (33.3)	0 (0.0)	3 (50.0)	5 (41.7)	1 (100)	1 (100)	3 (42.9)	1 (50.0)
Calcifications [*n* (%)]	0 (0.0)	0 (0.0)	1 (16.7)	0 (0.0)	0 (0.0)	0 (0.0)	2 (28.6)	0 (0.0)
Spine abnormalities [*n* (%)]	4 (66.7)	1 (50.0)	0 (0.0)	6 (50.0)	1 (100)	1 (100)	2 (28.6)	1 (50.0)

EMG/NCS = electromyogram/nerve conduction studies; N/A = not applicable; SNHL = sensorineural hearing loss.

^a^Data are presented as count and the percentage over the total number of patients with the abnormal test in each complementation group, unless otherwise stated.

EMG/NCS frequently yielded abnormal results in XPD (64.7%), XPG (50.0%) and XPA (14.3%) (Table 6 and Supplementary Table 16). Axonal neuropathies were the most common type of neuropathy in all groups with abnormal EMG/NCS (≥50.0%), with only three patients presenting with mixed neuropathies (two XPD patients and one XPG patient). Sensory and/or sensorimotor neuropathies were found in the different groups. Remarkably, there were no cases of isolated motor neuropathies. Most studies were classified as mild-moderate in all the groups.

Brain and/or spine MRI showed abnormalities in a high percentage of patients in groups XPB (100%), XPG (87.5%) and XPD (70.6%) (Table 6, Supplementary Table 17 and Supplementary Fig. 7). Interestingly, 27.3% of XPC subjects showed abnormal neuroimaging, including one case of a possible low-grade glioma, which has remained relatively unchanged over the follow-up period of the study. Atrophy was typically global (supratentorial and infratentorial) in most complementation groups. Cerebellar atrophy was also a common finding across groups (except for XPE and XPV). White matter changes are a relatively frequent finding in all complementation groups (except for XPB). Calcifications were a rare feature (one XPC patient and two XPG patients with XPG/Cockayne syndrome overlap).

### Genetic characterization of the xeroderma pigmentosum cohort

#### Effect of mutation severity on scale scores and age of onset for different events

Overall, 53.8% of the patients were homozygous for a given mutation. Homozygosity was especially frequent in XPA (95.2%) and XPC (72.7%). Pathogenic mutations found in the participants and their severity scores are summarized in Supplementary Table 18. Since XP is due to loss-of-function mutations, participants received mutation severity scores based on their less deleterious allele.

First, associations of mutation severity and the scale scores were examined. When the complementation group was not considered in the statistical models (unadjusted analysis), the mutation severity score was not associated with SARA total mean score or rate of progression (*P* = 0.749 and *P* = 0.671, respectively). Similar results were found for ADL total mean score and rate of progression (*P* = 0.322 and *P* = 0.874, respectively). However, in the models including complementation group (adjusted analysis), mutation severity did show statistically significant effects on mean scores and/or progression rates. Nonetheless, after controlling for mutation severity, the effects of complementation group on SARA and ADL total mean scores and rates of progression remained largely unchanged. This means that differences in the scores and rates are explained by the different complementation groups and, for subjects of the same complementation group, higher severity of the mutations is associated with worse scores and/or rates. Therefore, effects of mutation severity within four complementation groups (XPA, XPD, XPG and XPV) were investigated individually.

**Figure 2:**
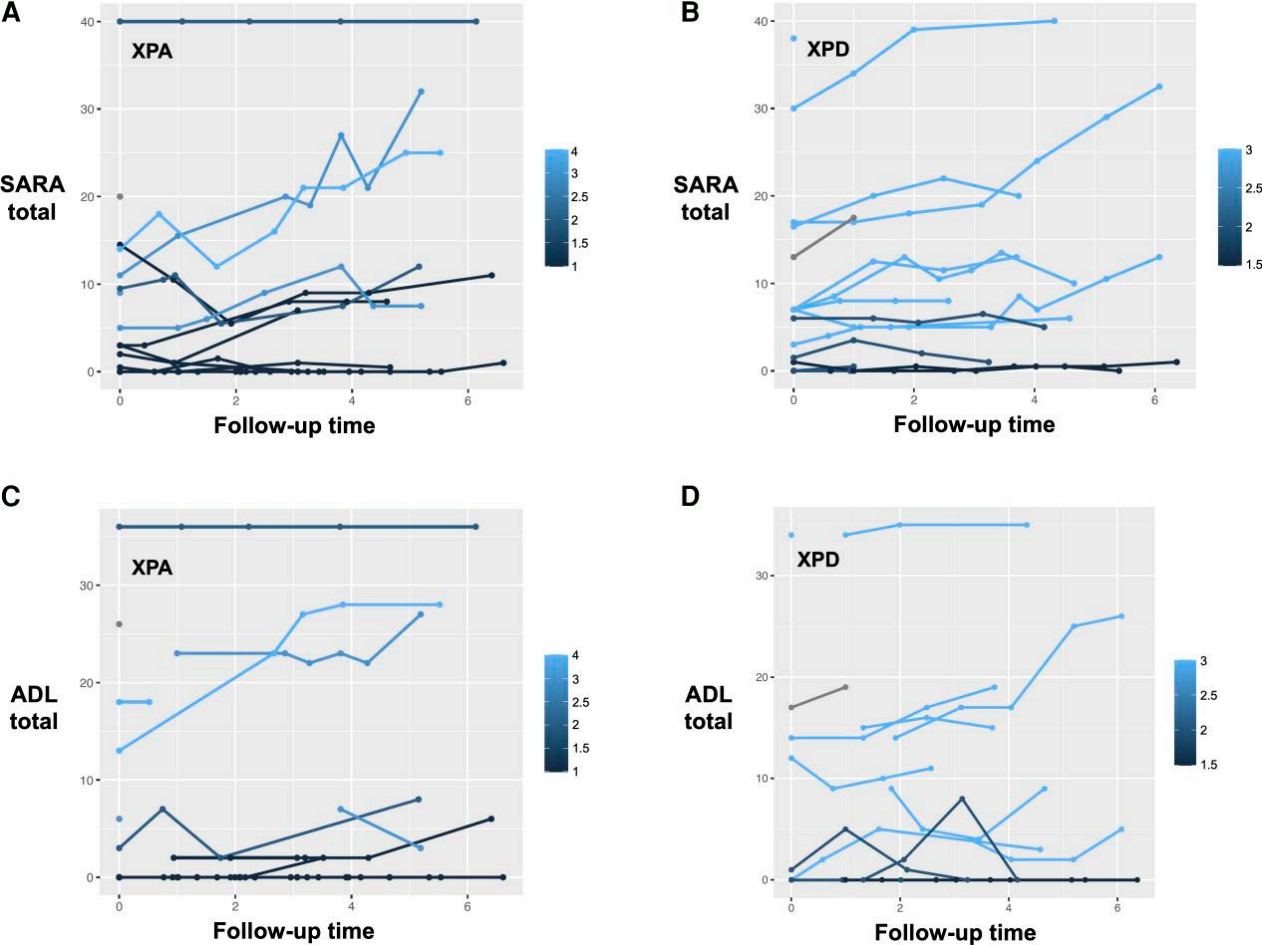
**Progression of clinical scores and patients’ mutation severity.** SARA total scores (points) over follow-up period (years), stratified by mutation severity scores, in XPA (**A**) and XPD (**B**). ADL total scores (points) over follow-up period (years), stratified by mutation severity scores, in XPA (**C**) and XPD (**D**). Each line represents an individual patient. Lighter colours correspond to more severe scores. ADL = Activities of Daily Living questionnaire; SARA = Scale for the Assessment and Rating of Ataxia.

For XPA and XPD, 1-unit increase in the rank of mutation severity was associated with statistically significant progression in SARA total scores (0.40 points/year, *P* = 0.002; and 0.60 points/year, *P* = 0.006, respectively) (Fig. 2A and B). Mutation severity was only associated with ADL total progression rate in XPA (0.35 points/year, per 1-unit increase in mutation severity rank, *P* = 0.002) (Fig. 2C), but not in XPD (*P* = 0.172) (Fig. 2D). For XPG and XPV, mutation severity was not associated with SARA or ADL progression rates.

Second, we analysed the association of mutation severity with age of onset for different events in the individual complementation groups. In XPA, there was a significant association between mutation severity and age of disease onset (lrank = 1.054, *P* = 0.003), age of diagnosis (lrank = 0.295, *P* = 0.05), age of first neurological signs (lrank = 0.531, *P* = 0.004), and age of first wheelchair use (lrank = 1.095, *P* = 0.05). Although neurological onset and first wheelchair use events were observed in XPD and XPG, sample sizes did not provide meaningful statistical power to test mutation severity associations. Sample size was also insufficient to demonstrate an association between mutation severity and age of disease onset in XPV.

## Discussion

In this study, we have performed a deep neurological phenotyping of a large heterogeneous cohort of patients diagnosed with XP, recruited in the UK National XP Service. As in previous studies in Europe and the USA,^1,6,17,44^ XPC and XPA were the most represented groups. XPB and XPF were extremely rare in our cohort. Multiple studies have shown that XPF patients may present with mild cutaneous symptoms and late-onset neurological syndromes.^19,21,25,26^ Since most patients were referred on the basis of their cutaneous symptoms, it is likely that a group of XPF patients is still undiagnosed^45^ and, therefore, could not be considered for the present study.

In our cohort, 38.7% of all patients presented with neurological symptoms. This higher proportion compared to other studies^1,7,24^ could be attributed to the group composition of our cohort, although our focus on neurological features and the careful phenotyping of these might have also played a role. The most frequent neurological symptoms at onset were imbalance, neurodevelopmental delay, cognitive symptoms and hearing impairment, which seem to be common in other series.^7,12,16,17^ XPA, XPD and XPG showed higher proportions of patients with neurological symptoms, in agreement with previous reports.^1,7,16-18^ None of our XPE or XPV patients had neurological complaints. XPC has been traditionally considered a group without neurological manifestations. However, several reports in the literature have described XPC patients with typical^17,30,44^ or atypical neurological presentations.^46,47^ The presence of consanguinity and, potentially, other recessive disorders has been suggested as an explanation for these atypical presentations. In our cohort 2/22 XPC patients (9.1%) presented with neurological symptoms, and consanguinity was reported only for one of the patients.

XP neurological disease has been classified into juvenile forms (onset before the age of 21) and adult forms (onset after 21 years of age).^48^ Surprisingly, there was a 20-year gap between both forms in the XPA, XPD and XPG groups in our cohort. These two forms could reflect different pathophysiological processes, i.e. a neurodevelopmental disorder with neurodegeneration, versus a pure neurodegenerative condition, as suggested for other genetic ataxias.^49-51^ In addition, this information is useful for prognostic purposes in XPA, XPD and XPG patients in our cohort.

There could be a sequential occurrence of cutaneous, ophthalmological and neurological symptoms in XPA, XPD and XPG, in agreement with previous data.^1,16^ This sequence may be related to the type of insult and the pathophysiology of the condition in the different tissues. The external UVR would have an immediate effect on the DNA of cells from the skin and the ocular surface. However, the pathophysiological process in the CNS could be more intricate. It is likely to result from the gradual accumulation of lesions generated by endogenous damaging agents, and the neurological symptoms are unlikely to manifest until a critical number of cells in the CNS have been lost as consequence of a failure to repair these lesions.

Several studies suggest that some types of oxidative DNA damage and, possibly, mitochondrial dysfunction are associated with the neurodegenerative process in XP. DNA lesions such as 8,5′-cyclopurine-2′-deoxynucleosides (cyclopurines) accumulate in XP neurons.^4,52-54^ These are likely candidates as causative agents of neurodegeneration in XP since cyclopurines are lesions that can only be repaired through NER, they are endogenously produced, chemically stable and they may block transcription processes.^52^ In brain tissue of XPA knockout mice (*Xpa^−^*^/−^), cyclopurines accumulated at a faster rate compared to wild-type animals.^53^ A role for mitochondrial dysfunction has also been proposed in recent studies. In XPA, persistent activation of the DNA damage response would cause a chronic activation of PARP1, leading to a decrease in NAD^+^ and SIRT1 activity and, subsequently, a defect in mitophagy.^55^

The XP neurological disease tends to present more frequently in some complementation groups than in others. This heterogeneity can be largely explained by the defective pathways in each genotype. Those groups with simultaneous impairment of GG-NER and TC-NER (XPA, XPB, XPD, XPF, XPG) would be more prone to experience neurodegeneration, as cells are more sensitive to killing by DNA damage when compared to the groups with preservation of TC-NER (XPC, XPE, XPV).^56^ In addition, there is some evidence that TC-NER is the crucial NER sub-pathway in differentiated neurons, as GG-NER activity has been found to be markedly reduced in these cells.^57,58^ Therefore, neurons carrying defects in TC-NER would show a higher vulnerability to oxidative DNA damage. In particular, large neurons such as Purkinje cells, neurons of the dorsal root ganglia and motor neurons seem to be especially susceptible, as they exhibit higher metabolic demands.^59,60^

Despite a similar frequency of skin cancers compared to previous reports,^1,7,11,44^ our cohort showed a later age of onset for these tumours. The improvement in prevention and treatment of skin cancers,^11,12,61^ and the lack of effective therapies for the neurological disease, will likely result in neurological complications becoming the main cause of mortality in XP patients, at least in countries with well developed healthcare systems.^1,7^

To our knowledge, this is the first study in which progression of the XP neurological disease has been systematically studied using rating instruments validated in other ataxic conditions. The motor function items of a previous proposed scale^11^ were not based on neurological signs or precise examination findings. In addition, the previous tool was devised for severe XPA patients, and it might not be relevant for milder forms of XP neurological disease.

Cerebellar ataxia is a common feature in XP neurological disease.^1,7,16,17,19^ We showed that SARA total scores were higher in XPA, XPD and XPG compared to XPC, XPE and XPV, confirming the presence of cerebellar signs in the former groups. Interestingly, XPV patients showed some mild kinetic tremor. We found significant worsening in SARA total scores for XPA and XPD, both groups progressing between 0.5 and 1 point per year. These rates are average estimates for each group as a whole, and therefore, higher rates could be expected in more homogeneous cohorts with severe patients.^12,16,34^ Progression rates were slower than those of SCA1 (2.11 points per year), SCA3 (1.56 points per year) and SCA2 (1.49 points per year), but similar to those of SCA6 (0.80 points per year)^62^ or Friedreich’s ataxia (0.77 points per year).^63^ Regarding individual items, SARA gait, SARA stance and SARA speech rates showed statistically significant measurable progression in XPA and XPD.

The presence of neurological disease is associated with increased disability. Hence, ADL total scores showed significantly higher levels of disability in XPA, XPD and XPG compared to XPC, XPE and XPV. Remarkably, ADL speech and ADL walking were among the most affected activities in XPA, XPD and XPG. Therefore, end points measuring gait and speech may be useful in future clinical trials, and sensitive assessments for these signs should be investigated in XP.

Patients’ complementation group was the main effect explaining SARA and ADL mean scores and progression rates. Notwithstanding this, we demonstrated that more severe mutations were associated with a faster progression in SARA total in XPA and XPD patients, and a faster progression in ADL total in XPA patients. In addition, in XPA, more severe mutations were associated with an earlier onset of the condition, an earlier diagnosis, an earlier onset of neurological features and an earlier onset of wheelchair use. Therefore, there are some genotype-phenotype associations in XPA and XPD. Previous studies have suggested that XPA patients with mutations closer to the 3′-end of the *XPA* gene present with milder phenotypes.^18,64^ Although the genotype-phenotype associations are not perfect, mutation severity could be used as a prognostic biomarker for stratification and enrichment purposes in future clinical trials.

Deep neurological phenotyping in all the complementation groups yielded some interesting results. Hyporeflexia and hypopallesthaesia were frequent in XPA, XPD and XPG, as they are associated with peripheral neuropathy. Surprisingly, XPV patients also presented with lower limb hyporeflexia (in 53.2% of cases) and impaired vibration sense (in 17.4% of cases). The prevalence of these signs seems to be higher in our XPV group than in a sample of healthy elderly population^65^ and, therefore, these signs could be part of the asymptomatic XP neurological disease that was previously reported in XPC cases.^30^ Another example of this would be the presence of kinetic tremor in the XPV group. Upper motor neuron signs were present in the groups with symptomatic XP neurological disease and, therefore, XP should be considered in the differential diagnosis of complicated spastic paraparesis.^66^ Muscle weakness was preferentially distal and in the lower limbs, being congruent with a neuropathic pattern.

Chorea and dystonia were frequent findings in patients with XP. Chorea has been previously reported in XPA,^17,19^ XPB,^27^ XPD,^19^ XPF^19,21,25,26^ and XPG^27^ and, therefore, XP should be included in the differential diagnosis of Huntington’s disease-like phenotypes. Interestingly, some adult XPC patients displayed upper limb dystonic postures elicited by Fogs’ feet-hands test. This sign is a type of non-homologous associated movement related to immaturity or disruption of motor control pathways.^67^ This could represent another instance of the asymptomatic XP neurological disease in XPC patients.

XPA, XPD and XPG frequently showed cerebellar and brainstem oculomotor signs, with preferential impairment of movements in the vertical plane rather than the horizontal plane.

SNHL is a common manifestation of the XP neurological disease.^1,16,17,19,24^ SNHL in XP shows a predominant cochlear component and limited pathological data in a XPA case suggests that loss of primary cochlear neurons would precede that of cochlear hairy cells.^24^ In our cohort, SNHL was found in 28 patients (30.1% of the total sample), and it is possible that some patients with subclinical hearing loss have not been identified, as PTA were done on a clinical basis. Remarkably, a few XPE and XPV patients also presented with mild SNHL. Although other causes for SNHL could not be ruled out, these findings could represent features of an asymptomatic XP neurological disease,^30^ as SNHL has also been previously reported in XPC and XPE patients without overt neurological manifestations.^24^ In a previous report, XP patients without neurological disease showed higher PTA thresholds compared to control subjects, but the rate of progression was similar in both groups, whereas both the PTA thresholds and rates of progression were higher in XP patients with neurological disease compared to control subjects.^24^

Peripheral neuropathy is one of the hallmarks of the XP neurological disease.^16,17,19,28,29^ In our cohort, abnormal EMG/NCS were mainly found in XPA, XPD and XPG. Typically, neuropathies were of the sensory or sensorimotor axonal type and contrary to previous reports,^29^ both types were found in XPA, XPD and XPG. None of the EMG/NCS showed an isolated motor neuropathy and, therefore, this finding would be atypical in XP. Previous studies have shown the slowly progressive length-dependent character of the peripheral neuropathy in XP, and its preference for large myelinated sensory fibres.^28,29,68-70^ The amplitude of the nerve action potentials was associated with other measures of neurodegeneration in different studies.^28,29^ Interestingly, central conduction times also seem impaired in XP patients, even more severely than the peripheral component.^20,22^

In our cohort, a total of 37 patients spanning all the complementation groups (39.8% of the total sample) showed abnormalities in their brain and/or spine MRI. In concordance with other studies,^17,19,31-33,71^ global atrophy (supratentorial and infratentorial) and cerebellar atrophy were common findings in patients’ brain MRI. Some patients in groups without overt neurological symptoms, especially in XPC, showed abnormal neuroradiological findings, in agreement with previous reports.^17,30^ This could be supportive of some underlying neurodegeneration in these patients. One of our XPC patients presented with an incidental intracranial lesion, possibly a low-grade glioma. XPC patients have showed predisposition to CNS tumours in other studies^14,15^ and, therefore, periodic neuroimaging testing would be advisable in these patients. Interestingly, abnormalities are not only limited to the brain parenchyma, as we also observed calvarial thickening and increased pneumatization of frontal sinuses in a XPD patient, findings previously reported only in individuals diagnosed with XPA.^17,31,33^

Our study has some limitations. XP is a very rare condition that comprises several genotypes. Therefore, the sample size of the different complementation groups was reduced, and this may have prevented our study from having enough statistical power to detect all clinically important differences among the groups, or to clarify the potentially confounding effect of different variables (e.g. age, time since onset). Patients with mild skin symptoms and late-onset neurological syndromes may have been under-represented. Our patients were recruited in different stages of the condition and it could be plausible that progression rates of the neurological disease vary along the disease course. Some of the measures were reported retrospectively and, therefore, a recall bias may exist. Our mutation severity score had some degree of subjectivity. The frequency of the abnormal findings in the ancillary tests may be underestimated, as we cannot rule out the presence of subclinical findings in subjects that did not receive these investigations. Finally, we cannot completely rule out that other comorbidities or factors are partially responsible for some of the findings on patients’ physical examination or ancillary tests. Abnormalities were presumed to be linked to XP when these were not atypical for the XP neurological disease.

In summary, we have characterized the XP neurological disease in a heterogenous group of patients. It is of the utmost importance to recognize the typical cutaneous and ophthalmological symptoms in patients with idiopathic late-onset neurological syndromes compatible with the XP neurological disease. While a full-blown neurological disease can appear in XPA, XPD and XPG patients, an asymptomatic XP neurological disease could be present in XPC, XPE and XPV patients. Walking and speech in XP patients were specially affected, and both warrant further investigation to explore their potential use as clinical outcomes. Patients’ mutation severity could be used as a prognostic biomarker for stratification purposes in future trials. XP patients should receive a multidisciplinary assessment and management, regardless of complementation group, including input from dermatology, ophthalmology, neurology, neuropsychology, clinical genetics, medical photography, specialized nursing and patient support groups.

## Supplementary Material

awad266_Supplementary_Data

## Data Availability

The data that support the findings of this study are available on reasonable request from the corresponding author. The data are not publicly available due to privacy or ethical restrictions.
