# Supplementary material for: Neurological disease in xeroderma pigmentosum: prospective cohort study of its features and progression
Source: Brain. 2023 Dec 1;146(12):5044–59. doi: 10.1093/brain/awad266 (PMC10690019; doi:10.1093/brain/awad266)
Supplement: awad266_Supplementary_Data [file awad266_supplementary_data.pdf]

Supplementary Table 1 Description of the mutation severity score used in this study.

Rank	Description
1	Likely to have a mild effect on protein function
1.5	Similar molecular defect to rank 2, but mutation shows more residual activity
2	Likely to have an intermediate effect on protein function (e.g., missense variants near the active site)
2.5	Predicted to have major effect on protein function, but functional assay shows some preserved function
3	Likely to have a major effect on protein function
4	Complete inactivation of the protein (frameshift, truncating or missense variants that inactivate the protein)

Supplementary Text 1. Brief description of the Lasso method.

In the Lasso method, several regression models are estimated by increasing constraints on predictors' coefficients, i.e, progressively decreasing the coefficients towards the null value. Such level of constraint is defined by a parameter designated as lambda (λ). For model selection, a cross-validation approach is used, fitting models iteratively with 4/5 of the data and testing their accuracy with the excluded 1/5. Two different criteria can be applied to select a model: a) optimal cross-validation criterium (λ of the model with the best cross-validation fit); b) 1-standard error (1-SE) criterium (the model with a 1-SE more stringent level of constraint than the optimal λ value is chosen) (Supplementary Fig. 1).

Once a model is selected, two rules can be used to calculate model accuracy, i.e., the proportion of correct classifications: a) classification by the highest probability (data is assigned to the group with the highest probability); b) classification by the maximum odds ratio (data is assigned to the group with the highest odds ratio). Coefficients are presented as log(odds) for membership in each group. A threshold of log(odds)= |±0.4| approximately corresponds to OR= 1.5 for group membership, defining clinically significant effects. For the models considering SARA and ADL items, these variables were previously standardised to allow for comparisons in their effects.

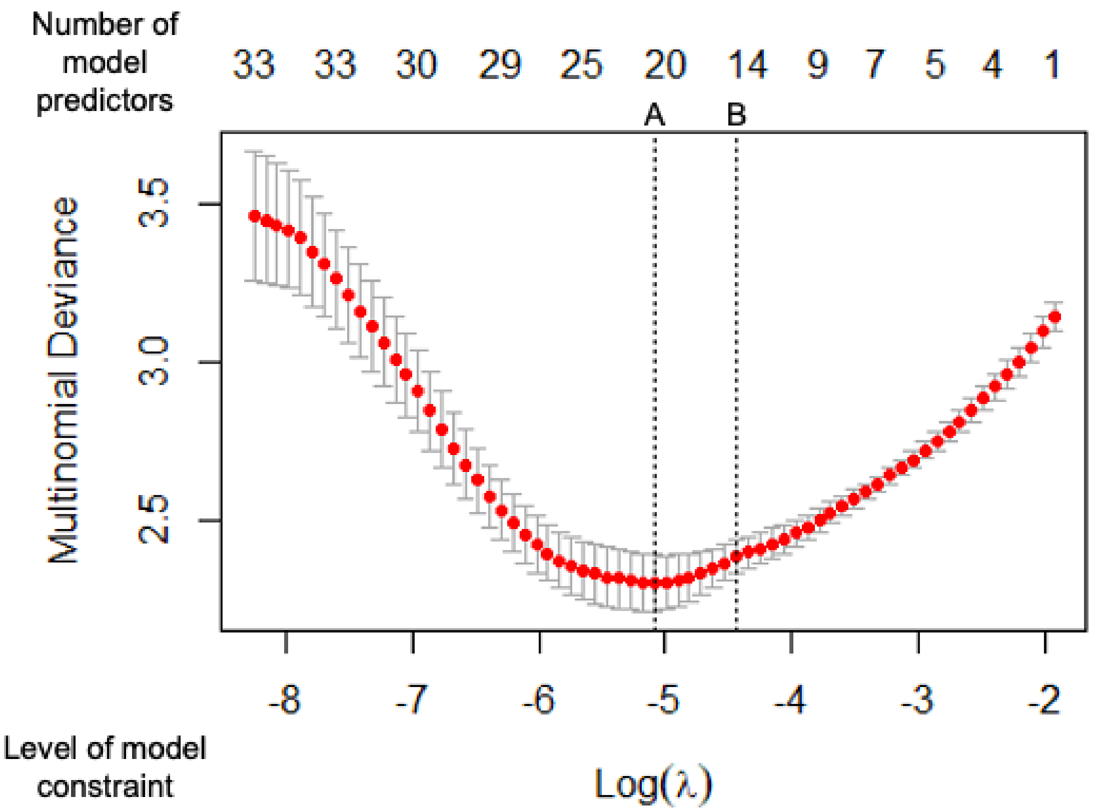

Supplementary Figure 1. Graphical representation of the criteria used for model selection in Lasso methods. The y-axis represents model multinomial deviance, that is, a measure of model goodness-of-fit. The top x-axis represents the number of predictors included in the model. The bottom x-axis represents the level of model constraint (λ value). **(A)** Model chosen with optimal cross-validation criterium. **(B)** Model chosen with 1-SE criterium. Image courtesy of Prof Douglas Langbehn.

Supplementary Table 2 Type of first symptom at onset for the different complementation groups[a]

	XPA	XPB	XPC	XPD	XPE	XPF	XPG	XPV	Total
Severe/ exaggerated/ easy sunburn [n (%)]	9 (42.9)	2 (100)	0 (0.0)	16 (94.1)	1 (14.3)	4 (100)	8 (100)	0 (0.0)	40 (43.0)
Lentigines [n (%)]	7 (33.3)	0 (0.0)	18 (81.8)	0 (0.0)	5 (71.4)	0 (0.0)	0 (0.0)	9 (75.0)	39 (41.9)
Basal cell carcinoma (BCC) [n (%)]	0 (0.0)	0 (0.0)	0 (0.0)	0 (0.0)	1 (14.3)	0 (0.0)	0 (0.0)	1 (8.3)	2 (2.2)
Malignant melanoma (MM) [n (%)]	0 (0.0)	0 (0.0)	0 (0.0)	0 (0.0)	0 (0.0)	0 (0.0)	0 (0.0)	2 (16.7)	2 (2.2)
Others [n (%)]	5 (23.8)	0 (0.0)	4 (18.2)	1 (5.9)	0 (0.0)	0 (0.0)	0 (0.0)	0 (0.0)	10 (10.8)

[a]Variables are expressed as number of subjects with percentages relative to each group size.

Others include: photophobia, conjunctival injection, hypopigmented macules, presymptomatic stage or combination of several symptoms.

Supplementary Table 3 Median diagnostic delay in the different complementation groups[a]

Complementation group	Age of disease onset (years, p50)	Age of diagnosis (years, p50)	Diagnostic delay (years)
XPA	2.5	13.5	11.0
XPB	0.5	36.5	36.0
XPC	2.5	4.5	2.0
XPD	0.5	3.5	3.0
XPE	12.5	41.5	29.0
XPF	2.0	9.0	7.0
XPG	0.5	8.5	8.0
XPV	18.0	45.5	27.5

[a]Diagnostic delay was calculated as p50 (diagnosis)– p50 (disease onset).

Supplementary Table 4 Frequency of first neurological symptoms in the different complementation groups[a]

	XPA	XPB	XPC	XPD	XPE	XPF	XPG	XPV	Total
Imbalance [n (%)]	4 (19.0)	0 (0.0)	1 (4.6)	3 (17.6)	0 (0.0)	0 (0.0)	0 (0.0)	0 (0.0)	8 (8.6)
Neurodevelopmental delay (motor, speech, intellectual) [n (%)]	2 (9.5)	0 (0.0)	1 (4.6)	3 (17.7)	0 (0.0)	0 (0.0)	1 (12.5)	0 (0.0)	7 (7.5)
Cognitive symptoms (behavioural, memory) [n (%)]	2 (9.5)	0 (0.0)	0 (0.0)	2 (11.8)	0 (0.0)	0 (0.0)	3 (37.5)	0 (0.0)	7 (7.5)
Hearing impairment [n (%)]	1 (4.8)	1 (50.0)	0 (0.0)	3 (17.7)	0 (0.0)	0 (0.0)	2 (25.0)	0 (0.0)	7 (7.5)
Cramps [n (%)]	0 (0.0)	0 (0.0)	0 (0.0)	1 (5.9)	0 (0.0)	0 (0.0)	0 (0.0)	0 (0.0)	1 (1.1)
Parkinsonism [n (%)]	1 (4.8)	0 (0.0)	0 (0.0)	0 (0.0)	0 (0.0)	0 (0.0)	0 (0.0)	0 (0.0)	1 (1.1)
Dyspraxia [n (%)]	0 (0.0)	0 (0.0)	0 (0.0)	1 (5.9)	0 (0.0)	1 (25.0)	0 (0.0)	0 (0.0)	2 (2.2)
No neurological symptoms [n (%)]	9 (42.9)	1 (50.0)	20 (90.9)	3 (17.6)	7 (100)	3 (75.0)	1 (12.5)	12 (100)	56 (60.2)

[a]Values represent number of subjects with percentages relative to the group size.

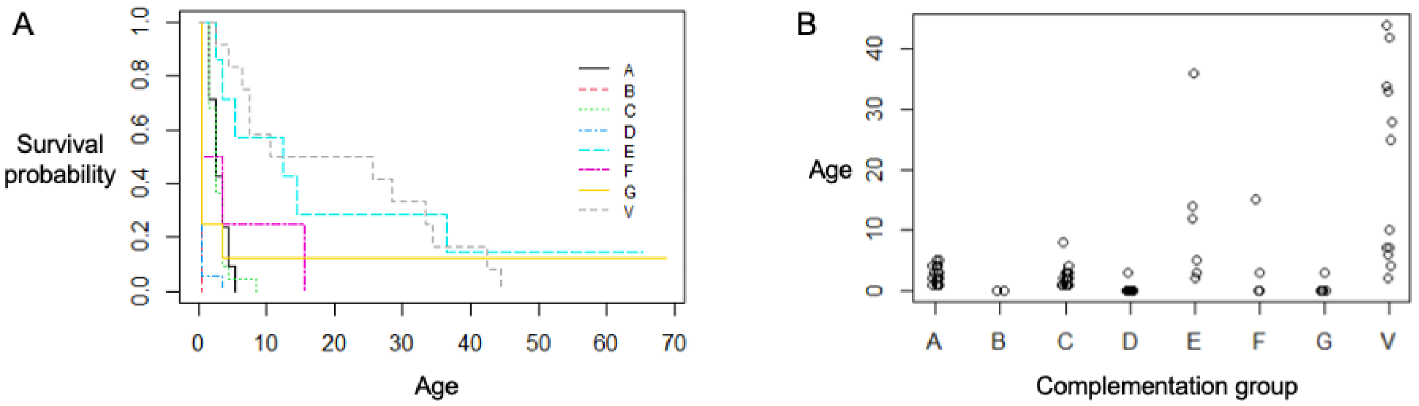

Supplementary Figure 2. Age of onset for skin symptoms in the different complementation groups. (A) Kaplan-Meier (KM) plot showing survival curves for each one of the groups (global log-rank test, χ^2= 65.1, df= 5, *P*< 0.001). **(B)** Dot plot representing individual values for age of onset of cutaneous symptoms in those subjects who presented the event of interest.

Supplementary Table 5 Type of first cutaneous symptoms for the different complementation groups[a]

	XPA	XPB	XPC	XPD	XPE	XPF	XPG	XPV	Total
Severe/ exaggerated/ easy sunburn [n (%)]	11 (52.4)	2 (100)	6 (27.3)	16 (94.1)	1 (14.3)	4 (100)	8 (100)	0 (0.0)	48 (51.6)
Lentigines [n (%)]	9 (42.9)	0 (0.0)	15 (68.2)	0 (0.0)	5 (71.4)	0 (0.0)	0 (0.0)	8 (66.7)	37 (39.8)
Basal cell carcinoma (BCC) [n (%)]	0 (0.0)	0 (0.0)	0 (0.0)	0 (0.0)	1 (14.3)	0 (0.0)	0 (0.0)	2 (16.7)	3 (3.2)
Malignant melanoma (MM) [n (%)]	0 (0.0)	0 (0.0)	0 (0.0)	0 (0.0)	0 (0.0)	0 (0.0)	0 (0.0)	2 (16.7)	2 (2.2)
Others [n (%)]	1 (4.8)	0 (0.0)	1 (4.6)	1 (5.9)	0 (0.0)	0 (0.0)	0 (0.0)	0 (0.0)	3 (3.2)

[a]Variables are expressed as number of subjects with percentages relative to group size.
Others include: subjects without cutaneous features and combination of different symptoms.

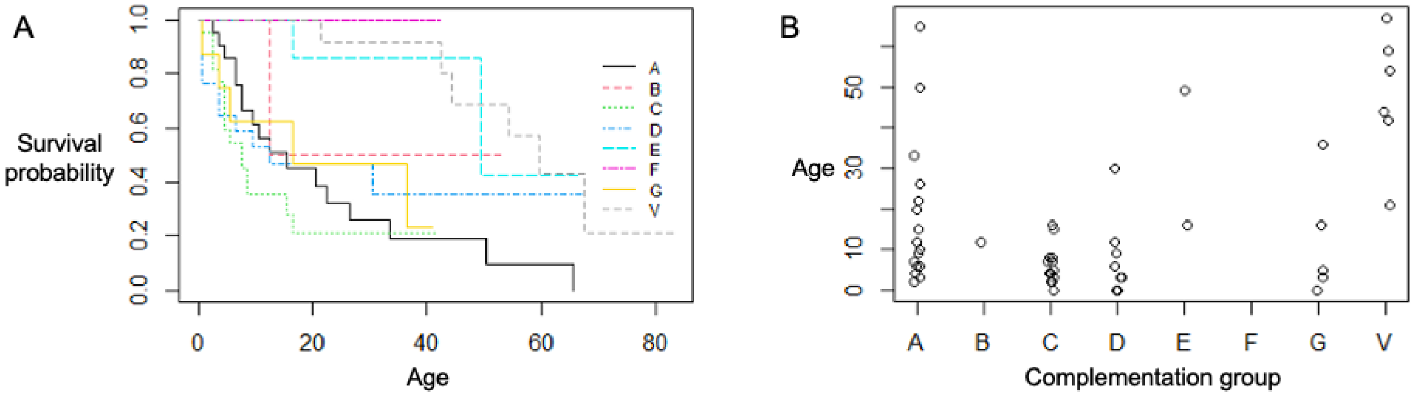

Supplementary Figure 3. Age of onset for eye symptoms in the different complementation groups. (A) Kaplan-Meier (KM) plot showing survival curves for each one of the groups (global log-rank test, χ^2= 21.0, df= 5, *P*< 0.001). **(B)** Dot plot representing individual values for age of onset of ocular symptoms in those subjects who presented the event of interest.

Supplementary Table 6 Type of first ophthalmological symptoms for the different complementation groups[a]

	XPA	XPB	XPC	XPD	XPE	XPF	XPG	XPV	Total
Photophobia [n (%)]	10 (47.6)	0 (0.0)	11 (50.0)	9 (52.9)	3 (42.9)	0 (0.0)	6 (75.0)	8 (66.7)	47 (50.5)
Pterygia [n (%)]	4 (19.1)	1 (50.0)	3 (13.6)	1 (5.9)	0 (0.0)	0 (0.0)	1 (12.5)	0 (0.0)	10 (10.8)
No ophthalmic symptoms [n (%)]	4 (19.0)	1 (50.0)	6 (27.3)	2 (11.8)	2 (28.6)	4 (100)	0 (0.0)	0 (0.0)	19 (20.4)
Others [n (%)]	3 (14.3)	0 (0.0)	1 (4.5)	4 (23.5)	1 (14.3)	0 (0.0)	1 (12.5)	4 (33.3)	14 (15.1)

[a]Variables are expressed as number of subjects with percentages relative to group size.
Others include: ocular surface cancer, conjunctival pigmentation, blepharitis, cicatricial ectropion, interpalpebral conjunctival melanosis, pinguecula, lagophthalmos/ectropion, keratopathy, and combination of symptoms.

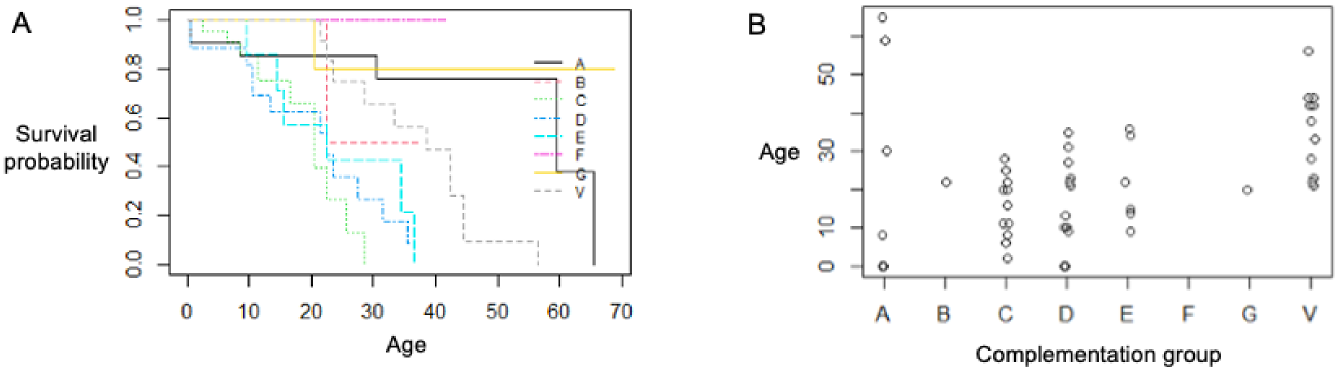

Supplementary Figure 4. Age of occurrence of the first cutaneous neoplasm in the different complementation groups. (A) Kaplan-Meier (KM) plot showing survival curves for each one of the groups (global log-rank test, χ^2= 28.1, df= 5, *P*< 0.001). **(B)** Dot plot representing individual values for age of onset of cutaneous neoplasms in those subjects who presented the event of interest.

Supplementary Table 7 Type of first cutaneous neoplasms for the different complementation groups[a]

	XPA	XPB	XPC	XPD	XPE	XPF	XPG	XPV	Total
Basal cell carcinoma (BCC) [n (%)]	2 (9.5)	0 (0.0)	4 (18.2)	8 (47.1)	3 (42.9)	0 (0.0)	1 (12.5)	5 (41.7)	23 (24.7)
Squamous cell carcinoma (SCC) [n (%)]	2 (9.5)	0 (0.0)	4 (18.2)	1 (5.9)	0 (0.0)	0 (0.0)	0 (0.0)	2 (16.7)	9 (9.7)
Melanoma in situ (MIS) [n (%)]	0 (0.0)	0 (0.0)	2 (9.1)	0 (0.0)	0 (0.0)	0 (0.0)	0 (0.0)	2 (16.7)	4 (4.9)
Malignant melanoma (MM) [n (%)]	1 (4.8)	1 (50.0)	1 (4.6)	0 (0.0)	1 (14.3)	0 (0.0)	0 (0.0)	2 (16.7)	6 (6.5)
No cutaneous cancer [n (%)]	14 (66.7)	1 (50.0)	11 (50.0)	5 (29.4)	1 (14.3)	4 (100)	7 (87.5)	1 (8.3)	44 (47.3)
Others [n (%)]	0 (0.0)	0 (0.0)	0 (0.0)	1 (5.9)	1 (14.3)	0 (0.0)	0 (0.0)	0 (0.0)	2 (2.2)

[a]Variables are expressed as number of subjects with percentages relative to group size.

Others include: multiple types of initial cutaneous cancer. In XPD, BCC and SCC. In XPE, BCC and MIS.

Supplementary Text 2. Description of the neuropathological findings in an XPD patient who passed away at the age of 28.

By gross examination, there was generalised cortical atrophy with marked ventricular dilatation (Supplementary Fig. 5A-5C). The head of the caudate, the thalamus, the limbic structures and the brainstem were markedly atrophic (Supplementary Fig. 5B and 5C, Supplementary Fig. 6B and 6C). The cranial nerves also showed marked atrophy by naked eye. Mild atrophy was also seen in the cerebellum with a small dentate nucleus (Supplementary Fig. 6A).

Histology confirmed thinning of the cortical ribbon with loss of pyramidal neurons and increased glial cells in the frontal, parietal and temporal lobes (Supplementary Fig. 5D). The occipital lobe also showed marked neuronal loss, with relative preservation of the calcarine cortex (Supplementary Fig. 5E). The amygdala and the hippocampus displayed severe neuronal loss, with moderate thinning of the dentate fascia (Supplementary Fig. 5F and 5G). All these areas showed loss of myelin in the white matter with gliosis. The corpus callosum was markedly thinned. The thalamus and the caudate nucleus showed marked neuronal loss, microvacuolation and gliosis (Supplementary Fig. 5H and 5I), whereas the globus pallidus, the putamen and the mammillary bodies were moderately affected. The claustrum was severely atrophic. There was prominent loss of pigmented neurons in the substantia nigra and locus coeruleus (Supplementary Fig. 6F). All brainstem nuclei, particularly the vestibulocochlear nuclei, showed severe neuronal loss (Supplementary Fig. 6G-6I). The Vth, VIIth and VIIIth cranial nerves were severely atrophic. In the Vth cranial nerve, myelin stain showed no obvious demyelination, with negative CD3 stain (for T-cells). CD68 revealed evenly distributed microglial cells and macrophages in the pons and the trigeminal nerve, respectively. Neurofilament stain (NF200KD) showed loss of axons. The overall features did not support acquired inflammatory demyelinating neuropathy. GFAP stain revealed mild

reactive and prominent fibrillary astrocytosis in the brainstem. The cerebellum presented with marked Purkinje cells loss associated with Bergmann's gliosis and some axonal torpedoes, and thinning of the granular cell layer (Supplementary Fig. 6D and 6E). The cerebellar white matter showed some atrophy and myelin loss, and marked neuronal loss was present in the dentate nucleus. Extensive immunohistochemistry was performed and showed negative results with β-amyloid, hyperphosphorylated tau, TDP43, p62 and α-synuclein, excluding all common neurodegenerative disorders.

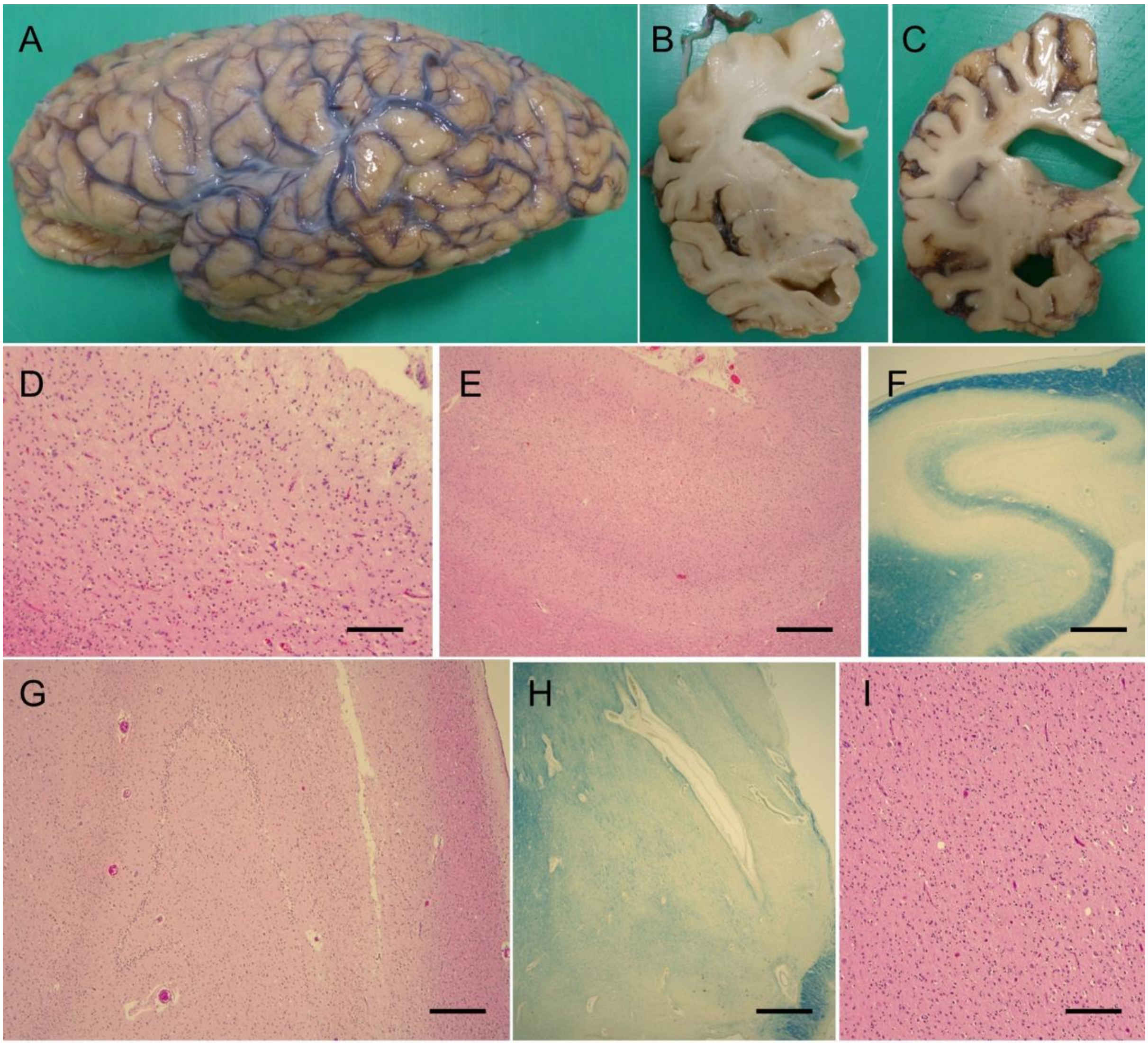

Supplementary Figure 5. Neuropathological findings in brain tissue of a patient diagnosed with xeroderma pigmentosum complementation group D (XPD), aged 28.

(A) Marked generalised cortical atrophy with thickened leptomeninges. **(B)-(C)** Coronal sectioning of the left half brain showing deep sulci, markedly dilated ventricles, and prominent atrophy of the caudate nucleus and the thalamus. The amygdala and the hippocampus are also small **(C)**. **(D)** Severe loss of neurones, particularly pyramidal cells, in the frontal cortex. **(E)** The calcarine cortex shows no obvious neuronal loss. **(F)-(G)** The hippocampus reveals subtotal neuronal loss, and the dentate fascia is also thinned. **(H)-(I)**

The thalamus shows atrophy and marked neuronal loss in higher magnification **(I)**. **(D)**, **(E)**, **(G)** and **(I)**: haematoxylin-eosin. **(F)** and **(H)**: Luxol fast blue-Nissl stain. Scale bars: **(D)**: 60μm; **(E)**, **(G)**: 150μm; **(F)**, **(H)**: 1mm; I: 80μm.

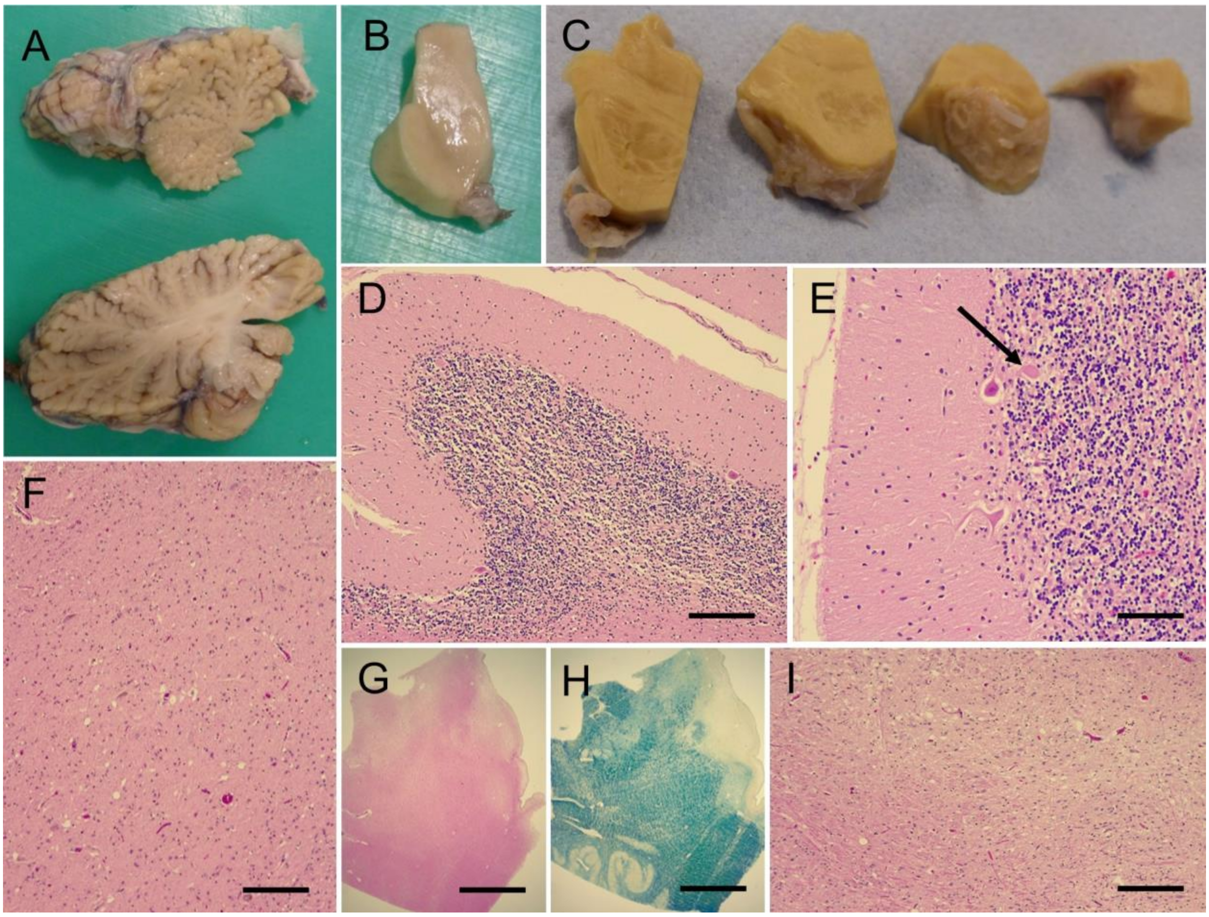

Supplementary Figure 6. Neuropathological findings in cerebellum and brainstem tissue of a patient diagnosed with xeroderma pigmentosum complementation group D (XPD), aged 28.

(A) Mild cerebellar cortical atrophy with small dentate nucleus. **(B)** Depigmentation of the substantia nigra in the midbrain. **(C)** Marked atrophy of the pons and the medulla oblongata. The cranial nerves are also very thin. **(D)** Severe loss of Purkinje cells and thinning of the granular cell layer. **(E)** Axonal torpedo (arrow) in the granular cell layer below a rare surviving Purkinje cell. **(F)** Hardly any surviving neurons in the substantia nigra. **(G)**-**(H)** Marked atrophy of the medulla oblongata. **(I)** Severe neuronal loss in the lateral vestibular nuclei by higher magnification. **(D)**-**(G)**, **(I)**: haematoxylin-eosin. **(H)**: Luxol fast blue-Nissl stain. Scale bars: **(D)**: 150μm; **(E)**, **(F)** and **(I)**: 80μm; **(G)** and **(H)**: 1mm.

Supplementary Table 8 Estimated SARA total mean progression rates as a function of follow-up time, time-since-onset of the first disease manifestation, and time-since-onset of the first neurological symptoms[a]

Complementation group	Progression rate per year of follow-up time (unadjusted)	Progression rate per year of follow-up time (adjusted)	Progression rate per year since onset (first disease symptom)	Progression rate per year since onset (neurological symptoms)
XPA	0.63 (0.38, 0.89)	0.63 (0.38, 0.89)	0.30 (0.08, 0.52)	0.56 (0.17, 0.96)
XPC	-0.03 (-0.26, 0.19)	-0.03 (-0.26, 0.19)	0.00 (-0.03, 0.03)	N/A
XPD	0.91 (0.61, 1.21)	0.91 (0.61, 1.20)	0.73 (0.46, 1.01)	1.04 (0.70, 1.38)
XPE	0.05 (-0.52, 0.62)	0.04 (-0.53, 0.61)	0.02 (-0.04, 0.08)	N/A
XPG	0.54 (-0.07, 1.15)	0.53 (-0.08, 1.15)	0.09 (-0.34, 0.52)	0.26 (-0.92, 1.45)
XPV	0.07 (-0.28, 0.43)	0.08 (-0.28, 0.44)	0.10 (0.03, 0.17)	N/A

[a]Results represent estimated mean progression rates for SARA total score in each complementation group (accounting for intrasubject variability), with their 95% CIs. The first two models represent the estimated mean progression rates per year of observed follow-up time (unadjusted and adjusted by baseline age, respectively). The third model corresponds to the estimates per year since onset of the first symptoms of the condition. The fourth model shows the estimates per year since onset of neurological symptoms. This last model only considered those subjects with self-reported onset of neurological manifestations and, therefore, estimates were not calculated in XPC, XPE and XPV, as most subjects in these groups did not refer an onset of neurological manifestations.
N/A: not applicable.

Supplementary Table 9 Longitudinal analysis of SARA items[a]

SARA item	XPA	XPC	XPD	XPE	XPG	XPV	Global χ^2 test	Statistically significant pairwise contrasts
SARA gait	0.13 (0.06, 0.19)	-0.01 (-0.07, 0.05)	0.21 (0.13, 0.29)	0.01 (-0.13, 0.16)	0.40 (0.24, 0.57)	0.01 (-0.08, 0.11)	38.19, df 5, *P*< 0.001	G vs A, C, E, V<br>D vs C, V<br>A vs C, G
SARA stance	0.19 (0.13, 0.25)	0.00 (-0.05, 0.05)	0.19 (0.12, 0.25)	-0.01 (-0.14, 0.11)	0.33 (0.20, 0.47)	0.01 (-0.07, 0.09)	46.89, df 5, *P*< 0.001	G vs C, E, V<br>A vs C, V<br>D vs C, V
SARA sitting	0.00 (-0.05, 0.04)	0.00 (-0.04, 0.04)	0.05 (0.00, 0.10)	0.00 (-0.09, 0.09)	0.08 (-0.03, 0.18)	0.00 (-0.06, 0.06)	5.01, df 5, *P*= 0.415	Non-significant
SARA speech	0.13 (0.07, 0.19)	0.00 (-0.06, 0.06)	0.14 (0.06, 0.21)	0.00 (-0.14, 0.14)	-0.36 (-0.51, -0.21)	0.02 (-0.07, 0.11)	42.98, df 5, *P*< 0.001	D vs C, G<br>A vs C, G<br>G vs A, C, D, E, V
SARA finger chase	-0.01 (-0.05, 0.02)	-0.01 (-0.04, 0.03)	0.06 (0.02, 0.10)	0.00 (-0.08, 0.08)	-0.04 (-0.13, 0.04)	-0.01 (-0.06, 0.05)	9.13, df 5, *P*= 0.104	Non-significant
SARA nose-finger	0.05 (0.00, 0.11)	-0.01 (-0.06, 0.04)	0.09 (0.03, 0.16)	0.05 (-0.07, 0.17)	0.05 (-0.08, 0.18)	0.05 (-0.03, 0.12)	7.04, df 5, *P*= 0.218	Non-significant
SARA fast alternating hand movements	0.06 (-0.00, 0.12)	0.00 (-0.05, 0.05)	0.03 (-0.04, 0.10)	0.00 (-0.13, 0.13)	0.06 (-0.08, 0.20)	-0.03 (-0.11, 0.05)	4.39, df 5, *P*= 0.493	Non-significant
SARA heel-shin	0.11 (0.04, 0.18)	0.00 (-0.06, 0.06)	0.09 (0.00, 0.17)	0.00 (-0.15, 0.16)	-0.09 (-0.26, 0.08)	0.01 (-0.09, 0.10)	10.14, df 5, *P*= 0.071	Non-significant
SARA total	0.63 (0.38, 0.89)	-0.03 (-0.26, 0.19)	0.91 (0.61, 1.21)	0.05 (-0.52, 0.62)	0.54 (-0.07, 1.15)	0.07 (-0.29, 0.43)	32.37, df 5, *P*< 0.001	D vs C, V<br>A vs C

[a]Results represent estimated mean progression rates (points per year), as a function of follow-up time, for each score in each complementation group (accounting for intrasubject variability), with their 95% CIs. χ^2 values represent the test for global differences among progression rates in the groups. If such test is significant (*P*< 0.05), pairwise contrasts are calculated and corrected for multiple comparisons via the Tukey's method. Statistically significant contrasts for each item (*P*< 0.05) are summarised in the last column.

Supplementary Table 10 Cross-sectional analysis of ADL items with repeated measures ANOVA with random groups effects[a]

ADL item	XPA	XPC	XPD	XPE	XPG	XPV	Global χ^2 test	Statistically significant pairwise contrasts
ADL speech	1.01 (0.55, 1.47)	0.03 (-0.43, 0.48)	1.82 (1.31, 2.33)	0.00 (-0.80, 0.80)	1.28 (0.54, 2.03)	0.00 (-0.61. 0.61)	35.13, df 5, *P*< 0.001	D vs C, E, V<br>A vs C
ADL swallowing	0.51 (0.15, 0.88)	0.00 (-0.36, 0.36)	0.91 (0.49, 1.33)	0.00 (-0.64, 0.64)	0.44 (-0.15, 1.04)	0.04 (-0.45, 0.53)	14.20, df 5, *P*= 0.014	D vs C
ADL use of cutlery	0.90 (0.42, 1.38)	0.00 (-0.48, 0.48)	1.46 (0.92, 1.99)	0.00 (-0.84, 0.84)	0.74 (-0.04, 1.53)	0.04 (-0.60, 0.68)	21.80, df 5, *P*< 0.001	D vs C, E, V
ADL dressing	0.91 (0.44, 1.39)	0.00 (-0.47, 0.47)	1.22 (0.68, 1.76)	0.00 (-0.82, 0.82)	0.81 (0.04, 1.57)	0.08 (-0.54, 0.71)	17.77, df 5, *P*= 0.003	D vs C
ADL personal hygiene	1.02 (0.53, 1.51)	0.00 (-0.49, 0.49)	1.32 (0.76, 1.89)	0.00 (-0.86, 0.86)	0.77 (-0.03, 1.57)	0.04 (-0.61, 0.70)	19.41, df 5, *P*= 0.002	D vs C, V
ADL falls	0.85 (0.41, 1.29)	0.03 (-0.41, 0.47)	1.20 (0.71, 1.69)	0.00 (-0.77, 0.77)	1.11 (0.39, 1.83)	0.00 (-0.59, 0.59)	21.21, df 5, *P*< 0.001	D vs C, V
ADL walking	0.91 (0.48, 1.34)	0.00 (-0.43, 0.43)	1.42 (0.94, 1.89)	0.07 (-0.68, 0.82)	1.22 (0.52, 1.91)	0.32 (-0.25, 0.89)	25.15, df 5, *P*< 0.001	D vs C, E, V<br>G vs C<br>A vs C
ADL sitting	0.37 (0.05, 0.68)	0.00 (-0.31, 0.31)	0.55 (0.20, 0.91)	0.00 (-0.54, 0.54)	0.38 (-0.13, 0.89)	0.00 (-0.41, 0.41)	8.68, df 5, *P*= 0.122	Non-significant
ADL bladder function	0.59 (0.18, 1.01)	0.00 (-0.41, 0.41)	0.67 (0.20, 1.13)	0.21 (-0.52, 0.94)	1.00 (0.32, 1.68)	0.16 (-0.39, 0.72)	10.10, df 5, *P*= 0.072	Non-significant
ADL total	7.08 (3.47, 10.69)	0.06 (-3.63, 3.75)	10.26 (6.13, 14.38)	0.29 (-5.97, 6.54)	7.87 (2.02, 13.72)	0.63 (-4.15, 5.41)	20.14, df 5, *P*= 0.001	D vs C, V

[a]Results represent estimated mean scores in each complementation group (accounting for intrasubject variability), with their 95% CIs. χ^2 values represent the test for global differences among the groups. If such test is significant ($P< 0.05$), pairwise contrasts are calculated and corrected for multiple comparisons via the Tukey's method. Statistically significant contrasts for each item ($P< 0.05$) are summarised in the last column.

Supplementary Table 11 Longitudinal analysis of ADL items[a]

ADL item	XPA	XPC	XPD	XPE	XPG	XPV	Global χ^2 test	Statistically significant pairwise contrasts
ADL speech	0.03 (-0.02, 0.08)	0.00 (-0.04, 0.05)	0.11 (0.05, 0.17)	0.00 (-0.10, 0.10)	0.01 (-0.13, 0.14)	0.00 (-0.08, 0.08)	10.05, df 5, *P*= 0.074	D vs C
ADL swallowing	0.06 (0.01, 0.11)	0.00 (-0.04, 0.04)	-0.04 (-0.10, 0.02)	0.00 (-0.11, 0.11)	0.09 (-0.04, 0.22)	0.01 (-0.07, 0.08)	8.84, df 5, *P*= 0.115	Non-significant
ADL use of cutlery	0.05 (-0.01, 0.10)	0.00 (-0.04, 0.04)	0.12 (0.04, 0.19)	0.00 (-0.12, 0.12)	0.05 (-0.10, 0.19)	0.00 (-0.09, 0.09)	8.26, df 5, *P*= 0.143	Non-significant (D vs C, *P*= 0.082)
ADL dressing	0.05 (0.00, 0.11)	0.00 (-0.04, 0.04)	0.13 (0.06, 0.20)	0.00 (-0.12, 0.12)	0.08 (-0.07, 0.22)	0.01 (-0.08, 0.10)	10.30, df 5, *P*= 0.067	D vs C
ADL personal hygiene	0.05 (0.00, 0.11)	0.00 (-0.05, 0.05)	0.12 (0.04, 0.19)	0.00 (-0.12, 0.12)	0.15 (0.00, 0.29)	0.00 (-0.09, 0.10)	10.00, df 5, *P*= 0.075	Non-significant (D vs C, *P*= 0.107)
ADL falls	0.02 (-0.04, 0.08)	-0.01 (-0.06, 0.04)	0.10 (0.03, 0.16)	0.00 (-0.13, 0.13)	-0.11 (-0.26, 0.04)	0.00 (-0.08, 0.08)	9.56, df 5, *P*= 0.089	Non-significant (D vs C, *P*= 0.118)
ADL walking	0.07 (0.02, 0.12)	0.00 (-0.04, 0.04)	0.16 (0.11, 0.21)	0.02 (-0.09, 0.12)	0.01 (-0.12, 0.14)	0.07 (0.00, 0.14)	24.88, df 5, *P*< 0.001	D vs C
ADL sitting	0.02 (-0.02, 0.06)	0.00 (-0.04, 0.04)	0.03 (-0.02, 0.08)	0.00 (-0.10, 0.10)	0.04 (-0.08, 0.15)	0.00 (-0.07, 0.07)	1.37, df 5, *P*= 0.927	Non-significant
ADL bladder function	0.06 (0.00, 0.12)	0.00 (-0.05, 0.05)	0.12 (0.04, 0.19)	0.04 (-0.09, 0.18)	-0.01 (-0.17, 0.15)	0.00 (-0.11, 0.10)	7.94, df 5, *P*= 0.160	Non-significant (D vs C, *P*= 0.115)
ADL total	0.42 (0.16, 0.67)	-0.01 (-0.22, 0.21)	0.55 (0.19, 0.92)	0.07 (-0.51, 0.65)	0.36 (-0.32, 1.04)	0.16 (-0.28, 0.60)	10.39, df 5, *P*= 0.065	Non-significant (D vs C, *P*= 0.097; A vs C, *P*= 0.131)

[a]Results represent estimated mean progression rates (points per year), as a function of follow-up time, for each score in each complementation group (accounting for intrasubject variability), with their 95% CIs. χ^2 values represent the test for global differences in progression rates among the groups. If such test is significant ($P< 0.05$), pairwise contrasts are calculated and corrected for multiple comparisons via the Tukey's method. Statistically significant contrasts for each item ($P< 0.05$) are summarised in the last column.

Supplementary Table 12 Estimated mean frequencies for all INAS items in the different complementation groups[a]

INAS item	XPA	XPC	XPD	XPE	XPG	XPV	Statistically significant contrasts (95% credible intervals)
UL hyporeflexia	54.3	5.7	74.2	15.4	30.2	23.4	D vs C, E, G, V<br>A vs C, E, V<br>G vs C
LL hyporeflexia	47.5	3.4	85.8	12.4	34.3	53.2	D vs A, C, E, G, V<br>V vs C, E<br>A vs C<br>G vs C
Hypopallesthaesia	16.7	3.5	37.1	12.1	7.5	17.4	D vs C, G<br>A vs C
UL hyperreflexia	2.0	9.6	0.2	0.0	22.7	0.0	G vs A, D, E, V<br>C vs D, V
LL hyperreflexia	3.1	14.1	11.3	0.0	41.4	0.2	G vs A, D, E, V
Babinski sign	10.6	0.6	25.8	0.0	56.2	0.1	G vs A, C, E, V<br>D vs C, E, V<br>A vs C
Gait spasticity	15.7	4.1	19.5	0.0	24.2	0.1	G vs C, E, V<br>D vs E, V<br>A vs E, V
UL spasticity	8.0	0.4	8.2	0.0	9.5	0.0	D vs C<br>A vs C
LL spasticity	20.7	7.4	28.3	0.0	30.7	0.1	G vs C, E, V<br>D vs C, E, V<br>A vs E, V
Bulbar paresis	10.2	0.8	11.5	0.0	12.9	7.8	D vs C<br>A vs C
Proximal UL paresis	4.9	1.1	4.4	0.0	13.9	7.6	Non-credible
Distal UL paresis	9.9	0.7	13.8	0.0	27.6	20.3	G vs C, E<br>V vs C, E<br>D vs C<br>A vs C
Proximal LL paresis	10.1	1.0	12.1	0.0	8.0	7.5	Non-credible
Distal LL paresis	12.8	0.3	24.8	0.0	29.8	3.5	G vs C, E, V<br>D vs C, E, V<br>A vs C, E
Bulbar atrophy	4.0	0.5	2.6	0.0	21.2	3.8	G vs C, E
Proximal UL atrophy	4.8	0.9	3.4	0.0	14.0	7.0	Non-credible
Distal UL atrophy	7.0	0.8	9.1	0.0	17.1	6.8	G vs C
Proximal LL atrophy	4.2	0.6	2.9	0.0	17.0	4.0	G vs C, E
Distal LL atrophy	11.8	0.5	20.1	0.0	28.5	6.7	G vs C, E<br>D vs C, E<br>A vs C, E
Bulbar fasciculations	1.2	0.2	1.5	0.0	6.2	0.0	G vs C
UL fasciculations	0.2	0.3	1.7	0.0	0.0	0.0	Non-credible
LL fasciculations	0.2	0.3	0.1	0.0	0.0	0.0	Non-credible
Craniocervical chorea	3.2	0.4	11.2	0.0	32.8	0.0	G vs A, C, E, V<br>D vs C, E, V
Axial chorea	1.2	0.2	1.6	0.0	7.3	0.0	G vs C, V
UL chorea	5.8	0.4	23.1	0.0	34.7	0.0	G vs A, C, E, V<br>D vs A, C, E, V
LL chorea	1.2	0.2	3.5	0.0	11.3	0.0	G vs A, C, E, V

Craniocervical dystonia	9.5	0.3	7.0	0.0	31.4	0.0	G vs A, C, D, E, V<br>A vs C, E, V<br>D vs C
Axial dystonia	6.9	1.1	5.8	0.0	0.0	0.0	A vs E, G, V
UL dystonia	13.3	8.7	22.7	5.4	20.9	0.0	G vs V<br>D vs V<br>A vs V
LL dystonia	2.5	0.2	7.9	0.0	0.0	0.0	D vs C, G, V
Axial myoclonus	1.2	0.2	0.1	18.3	6.2	2.6	E vs A, C, D
UL myoclonus	3.2	1.0	5.1	4.0	2.9	0.0	D vs V
LL myoclonus	1.2	0.2	1.6	0.0	3.0	0.0	Non-credible
Axial rigidity	5.4	3.0	5.4	0.0	13.2	0.0	Non-credible
UL rigidity	8.9	5.0	3.6	0.0	9.7	7.7	Non-credible
LL rigidity	9.8	0.3	2.0	0.0	7.2	3.4	A vs C, E
Resting tremor	1.3	1.3	4.0	0.0	0.0	5.5	Non-credible
Altered ocular pursuit	34.3	1.7	49.8	0.0	46.0	18.4	D vs C, E, V<br>G vs C, E<br>A vs C, E<br>V vs C, E
Square wave jerks	1.3	0.2	6.7	0.0	15.6	0.0	G vs A, C, E, V<br>D vs C
Downbeat nystagmus	0.2	0.3	0.1	0.0	2.9	0.0	Non-credible
Gaze-evoked nystagmus on horizontal gaze	8.8	0.1	12.0	0.0	31.9	9.1	G vs A, C, E<br>D vs C, E<br>V vs C, E<br>A vs C, E
Gaze-evoked nystagmus on vertical gaze.	2.4	0.3	0.1	0.0	0.0	0.1	Non-credible
Hypometric saccades	25.5	7.9	40.8	0.0	48.8	16.4	G vs C, E<br>D vs C, E<br>A vs C, E<br>V vs E
Hypermetric saccades	0.1	1.1	18.8	0.0	10.7	5.8	D vs A, C, E<br>G vs A, C, E<br>V vs A
Horizontal ophthalmoparesis	9.3	1.0	23.0	0.0	0.1	0.1	D vs C, E, G, V
Vertical ophthalmoparesis	27.8	1.8	31.9	0.0	32.2	7.7	G vs C, E<br>D vs C, E<br>A vs C, E
Slowness of saccades	21.2	4.1	36.9	0.0	26.7	0.0	D vs C, E, V<br>G vs C, E, V<br>A vs C, E, V
Diplopia	4.3	0.6	13.8	0.0	0.0	0.0	D vs C, E, G, V
Urinary dysfunction	17.2	8.5	27.2	0.0	38.1	12.7	G vs C, E<br>D vs E
Cognitive impairment	58.4	17.6	85.0	15.1	83.2	4.3	D vs C, E, V<br>G vs C, E, V<br>A vs C, E, V
Vertigo	0.2	0.3	2.0	0.0	0.0	3.1	Non-credible
Problems with handwriting	9.3	1.1	45.7	0.0	35.4	0.1	D vs A, C, E, V<br>G vs C, E, V
Cramps	13.5	7.2	34.3	14.1	41.6	0.3	G vs C, V<br>D vs C, V

[a]Frequencies are presented as percentages. Frequencies were calculated using Bayesian methods to account for intrasubject variability. LL: lower limb. UL: upper limb.

Supplementary Table 13 Group classification calculated with the maximum odds ratio rule for the 1-SE cross-validated Lasso model for a reduced set of INAS items[a]

		Predicted group					
		XPA	XPC	XPD	XPE	XPG	XPV
True group	XPA	10	36	29	0	2	0
	XPC	3	83	1	0	9	0
	XPD	1	4	30	0	1	3
	XPE	1	14	1	0	0	1
	XPG	1	2	3	0	8	0
	XPV	0	13	3	0	0	12

[a]Data is presented as number of visits classified in a certain group (predicted group) and the group they truly belong to (true group).

Supplementary Table 14 Multinomial logistic regression coefficients of the 1-SE Lasso model for SARA and ADL items[a]

**Items**	**XPA**	**XPC**	**XPD**	**XPE**	**XPG**	**XPV**
SARA gait	0.13	-0.30	0.14	-0.09	0.23	-0.12
SARA speech	0.11	-0.14	0.20	-0.07	-0.03	-0.07
SARA nose-finger	0.00	-0.28	0.01	0.02	0.04	0.21
SARA heel-shin	0.03	-0.03	-0.01	-0.01	0.03	-0.02
ADL speech	0.10	-0.14	0.18	-0.09	0.07	-0.13
ADL personal hygiene	0.04	-0.01	0.00	-0.01	-0.01	-0.01
ADL sitting	0.04	0.00	-0.02	0.00	-0.01	0.00

[a]Coefficients are presented as log(odds). A positive coefficient (> 0) indicates that higher values in a predictor will increase the odds of being classified in that complementation group. A negative coefficient (< 0) indicates that higher values in a predictor will decrease the odds of being classified in that group. A coefficient close to the null value means that the value of an item in a complementation group is close to the average value of the other groups.

Supplementary Table 15 Characterisation of pure tone audiometry (PTA) tests in XP patients[a]

	XPA	XPB	XPC	XPD	XPE	XPF	XPG	XPV
Tested subjects [n, (% over total group size)]	14 (66.7)	2 (100)	13 (59.1)	15 (88.2)	2 (28.6)	2 (50.0)	7 (87.5)	5 (41.7)
Subjects with abnormal audiogram [n, (% over total group size)]	6 (28.6)	2 (100)	1 (4.5)	12 (70.6)	2 (28.6)	0 (0.0)	6 (75.0)	4 (33.3)
**Hearing loss type**								
SNHL [n (%)]	6 (100)	2 (100)	0 (0.0)	11 (91.7)	2 (100)	N/A	4 (66.7)	3 (75.0)
Conductive [n (%)]	0 (0.0)	0 (0.0)	1 (100)	0 (0.0)	0 (0.0)	N/A	1 (16.7)	0 (0.0)
Mixed [n (%)]	0 (0.0)	0 (0.0)	0 (0.0)	1 (8.3)	0 (0.0)	N/A	0 (0.0)	0 (0.0)
**Hearing loss severity**								
Mild [n (%)]	2 (33.3)	0 (0.0)	0 (0.0)	4 (33.3)	2 (100)	N/A	1 (16.7)	3 (75.0)
Moderate [n (%)]	3 (50.0)	0 (0.0)	1 (100)	6 (50.0)	0 (0.0)	N/A	2 (33.3)	1 (25.0)
Severe [n (%)]	1 (16.7)	2 (100)	0 (0.0)	1 (8.3)	0 (0.0)	N/A	3 (50.0)	0 (0.0)

[a]Data is presented as count, and the percentage over the total number of patients with the abnormal test in each complementation group, unless otherwise stated.
SNHL: sensorineural hearing loss. N/A: not applicable.

Supplementary Table 16 Characterisation of neurophysiological tests (EMG/NCS) in XP patients[a]

	XPA	XPB	XPC	XPD	XPE	XPF	XPG	XPV
Tested subjects [n (% over total group size)]	11 (52.4)	1 (50.0)	7 (31.8)	15 (88.2)	1 (14.3)	3 (75.0)	7 (87.5)	2 (16.7)
Subjects with abnormal EMG/NCS [n (% over total group size)]	3 (14.3)	0 (0.0)	1 (4.5)	11 (64.7)	0 (0.0)	0 (0.0)	4 (50.0)	1 (8.3)
**Type of neuropathy**								
Axonal [n (%)]	2 (66.7)	N/A	0 (0.0)	7 (63.6)	N/A	N/A	3 (75.0)	1 (100)
Demyelinating [n (%)]	0 (0.0)	N/A	0 (0.0)	0 (0.0)	N/A	N/A	0 (0.0)	0 (0.0)
Mixed [n (%)]	0 (0.0)	N/A	0 (0.0)	2 (18.2)	N/A	N/A	1 (25.0)	0 (0.0)
**Type of affected fibres**								
Motor [n (%)]	0 (0.0)	N/A	0 (0.0)	0 (0.0)	N/A	N/A	0 (0.0)	0 (0.0)
Sensory [n (%)]	2 (66.7)	N/A	1 (100)	8 (72.7)	N/A	N/A	2 (50.0)	0 (0.0)
Sensorimotor [n (%)]	1 (33.3)	N/A	0 (0.0)	3 (27.3)	N/A	N/A	2 (50.0)	1 (100)
**Severity**								
Mild [n (%)]	0 (0.0)	N/A	0 (0.0)	3 (27.3)	N/A	N/A	3 (75.0)	1 (100)
Moderate [n (%)]	2 (66.7)	N/A	0 (0.0)	4 (36.4)	N/A	N/A	0 (0.0)	0 (0.0)
Severe [n (%)]	1 (33.3)	N/A	0 (0.0)	1 (9.1)	N/A	N/A	1 (25.0)	0 (0.0)

[a]Data is presented as count, and the percentage over the total number of patients with the abnormal test in each complementation group, unless otherwise stated.
EMG/NCS: electromyogram/nerve conduction studies. N/A: not applicable.

Supplementary Table 17 Characterisation of imaging tests in XP patients[a]

	XPA	XPB	XPC	XPD	XPE	XPF	XPG	XPV
Tested subjects [n (% over total group size)]	12 (57.1)	2 (100)	10 (45.5)	14 (82.4)	1 (14.3)	4 (100)	8 (100)	3 (25.0)
Subjects with abnormal MRI [n (% over total group size)]	6 (28.6)	2 (100)	6 (27.3)	12 (70.6)	1 (14.3)	1 (25.0)	7 (87.5)	2 (16.7)
**Findings**								
Isolated infratentorial atrophy [n (%)]	1 (16.7)	0 (0.0)	1 (16.7)	0 (0.0)	0 (0.0)	0 (0.0)	0 (0.0)	0 (0.0)
Isolated supratentorial atrophy [n (%)]	0 (0.00)	0 (0.0)	1 (16.7)	3 (25.0)	0 (0.0)	0 (0.0)	0 (0.0)	0 (0.0)
Global atrophy [n (%)]	5 (83.3)	2 (100)	1 (16.7)	8 (66.7)	0 (0.0)	1 (100)	3 (42.9)	1 (50.0)
Cerebellar atrophy [n (%)]	5 (83.3)	2 (100)	2 (33.3)	7 (58.3)	0 (0.0)	1 (100)	2 (28.6)	0 (0.0)
Focal lesion [n (%)]	2 (33.3)	0 (0.0)	2 (33.3)	1 (8.3)	0 (0.0)	0 (0.0)	1 (14.3)	2 (100)
Delayed myelination [n (%)]	0 (0.0)	0 (0.0)	0 (0.0)	0 (0.0)	0 (0.0)	0 (0.0)	1 (14.3)	0 (0.0)
White matter changes [n (%)]	2 (33.3)	0 (0.0)	3 (50.0)	5 (41.7)	1 (100)	1 (100)	3 (42.9)	1 (50.0)
Calcifications [n (%)]	0 (0.0)	0 (0.0)	1 (16.7)	0 (0.0)	0 (0.0)	0 (0.0)	2 (28.6)	0 (0.0)
Spine abnormalities [n (%)]	4 (66.7)	1 (50.0)	0 (0.0)	6 (50.0)	1 (100)	1 (100)	2 (28.6)	1 (50.0)

[a]Data is presented as count, and the percentage over the total number of patients with the abnormal test in each complementation group, unless otherwise stated.

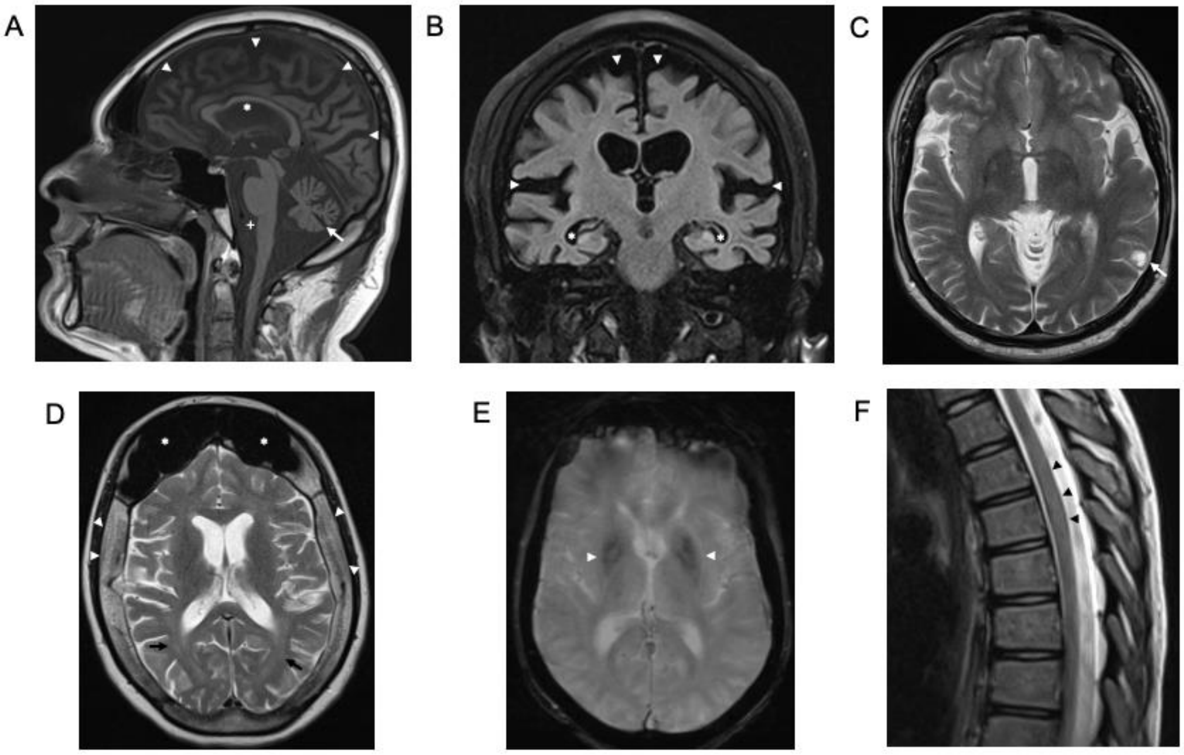

Supplementary Figure 7. MRI in XP patients. (A) Sagittal T1WI brain MRI in a 28-years-old XPA patient. Marked cortical (white arrowheads), cerebellar (white arrow) and brainstem (white cross) atrophy can be observed. The corpus callosum was slender (white asterisk). **(B)** Coronal T2WI FLAIR brain MRI in the same patient as in **(A)**. Bilateral hippocampal atrophy (white asterisks) and cortical atrophy (white arrows) are observed. **(C)** Axial T2WI brain MRI in a 24-years-old XPC patient. There is a hyperintense space-occupying lesion in the posterolateral temporal lobe (white arrow), which possibly represent a low-grade glioma. This lesion has remained relatively unchanged for four years. **(D)** Axial T2WI brain MRI in a 25-years-old XPD patient. There are diffuse changes in the posterior white matter (black arrows). Of note, calvarial thickening (white arrowheads) and marked pneumatisation of the frontal sinuses (white asterisks) can be observed. **(E)** Axial gradient echo T2*WI brain MRI in a 19-years-old XPG/CS patient. Abnormal mineralisation in the basal ganglia can be observed (white arrowheads). **(F)** Sagittal T2WI spine MRI in an 11-years-old XPD patient.

There is subtle hyperintensity at the T4-T6 level, with mild cord expansion (black arrowheads).

Supplementary Table 18 List of pathogenic mutations found in XP participants.

Group	cDNA sequence	Protein mutation	Absolute allele frequency	Relative allele frequency (%)	Severity score	Comments
**XPA**						
	c.555+8A>G	Splice	24	57.1	1	5% of normal protein made
	c.314G>A	p.Cys105Tyr	4	9.5	3	Missense mutation in critical Cys residue
	c.682C>T	p.Arg228X	4	9.5	2	Truncating variant near C-terminus with some possible activity
	c.266_267dupAA	p.Val90fs	2	4.8	4	Frameshift resulting in truncation
	c.389G>A	p.Arg130Lys	2	4.8	2	Conservative missense (some activity)
	c.640dupA	p.Met214fs	2	4.8	4	Frameshift resulting in truncation
	c.648_649delGA	p.Lys217fs	2	4.8	4	Frameshift resulting in truncation
	c.555G>C	p.Arg185His	1	2.4	3	Mis-splicing with undetectable UDS
	c.619C>T	p.Arg207X	1	2.4	4	Truncation
**XPB**						
	c.296T>C	p.Phe99Ser	3	75.0	Undetermined	
	c.1273C>T	p.Arg425X	1	25.0	4	Truncation
**XPC**						
	c.1243C>T	p.Arg415X	15	35.7	4	Truncation
	c.2176_2192del17	p.Glu726fs	9	21.4	4	Truncation
	c.1754A>G	p.Tyr585Cys	2	4.8	2	Conservative change, with significant remaining UDS
	c.1808G>A	p.Trp603X	2	4.8	4	Truncation
	c.2033+5G>A	Splice	2	4.8	2	Small amount of normal protein possibly made
	c.2251-1G>C	Splice	2	4.8	4	Mis-splicing – no evidence of residual activity
	c.2420+2T>C	Splice	2	4.8	4	Mis-splicing – no evidence of residual activity
	c.658C>T	p.Arg220X	2	4.8	4	Truncation
	c.877C>T	p.Arg293X	2	4.8	4	Truncation
	c.EX1del	Exon 1 deletion	1	2.4	4	Mis-splicing – no evidence of residual activity
	c.1021G>T	p.Ala341Ser	1	2.4	Undetermined	
	c.1111delA	p.Thr371fs	1	2.4	4	Truncation
	c.924_938del15	p.Leu309_313del	1	2.4	4	Truncation
**XPD**						
	c.2047C>T	p.Arg683Trp	14	43.8	3	Mutation produces big distortion
	c.1381C>G, c.2150C>G	p.Leu461Val; p.Ala717Gly	4	12.5	2	Low levels of full-length protein probably with residual activity
	c.1847G>A	p.Arg616Pro	2	6.3	4	Defective in transcription, resulting in lethality[a,b]
	c.2048G>A	p.Arg683Gln	2	6.3	1.5	More conservative change than Arg683Trp
	c.816-2A>G	Splice	2	6.3	4	Mis-splicing – no evidence of residual activity
	c.1378-26_1383del32	Splice	1	3.1	4	Mis-splicing – no evidence of residual activity

	c.1532G>A	p.Arg511Gln	1	3.1	Undetermined	
	c.1827delC	p.Phe610fs	1	3.1	4	Truncation
	c.1867dupG	p.Val623fs	1	3.1	4	Truncation
	c.1933_1934delCA	p.Gln645fs	1	3.1	4	Truncation
	c.1996C>T	p.Arg666Trp	1	3.1	Undetermined	
	c.335G>A	p.Arg112His	1	3.1	Undetermined	
	c.718+1C>A	Splice	1	3.1	4	Mis-splicing – no evidence of residual activity
**XPE**						
	c.459delT	p.Ile153fs	3	21.4	4	Truncation
	c.820C>T	p.Gln274X	3	21.4	4	Truncation
	c.1149delG	p.Met383fs	2	14.3	4	Truncation
	c.161G>A	p.Trp54X	2	14.3	4	Truncation
	c.457-2A>C	Splice	2	14.3	4	Mis-splicing – no evidence of residual activity
	c.487_488delAA	p.Lys163fs	1	7.1	4	Truncation
	c.973_984del12	p.Gly325_Pro328del	1	7.1	4	Truncation
**XPF**						
	c.1135C>T	p.Pro379Ser	4	50.0	1	This variant is listed in SNP databases, with an allele frequency of 0.3%
	c.1765C>T	p.Arg589Trp	2	25.0	4	Shown to have no activity[c]
	c.2395C>T	p.Arg799Trp	1	12.5	2	Strong effects on DNA repair
	c.872T>A	p.Leu291X	1	12.5	4	Truncation
**XPG**						
	c.264+1delG	Splice	4	25.0	2.5	Small amount of mutant protein made
	c.2453C>T	p.Ala818Val	3	18.8	2	Missense variant near the active site
	c.136delC	p.His46fs	2	12.5	4	Truncation
	c.1753G>T	p.Glu585X	2	12.5	4	Truncation
	c.869T>A	p.Ile290Asn	2	12.5	2	Based on conservation of the amino acid
	c.1842delT	p.Leu615fs	1	6.3	4	Truncation
	c.2383G>A	p.Ala795Thr	1	6.3	2	Missense variant near active site
	c.2586_2587delTA	p.Thr863fs	1	6.3	4	Truncation
**XPV**						
	c.1222_1225delACTT	p.Thr408fs	3	12.5	4	Truncation
	c.1066C>T	p.Arg356X	2	8.3	4	Truncation
	c.1117C>T	p.Gln373X	2	8.3	4	Truncation
	c.25G>T	p.Val9Phe	2	8.3	Undetermined	
	c.332G>A	p.Arg111His	2	8.3	3	Based on structural analysis[d]
	c.437dupA	p.Tyr146X	2	8.3	4	Truncation
	c.490+3A>G	Splice	2	8.3	Undetermined	
	c.681T>G	p.Cys227Trp	2	8.3	Undetermined	
	c.738delC	p.Phe247fs	2	8.3	4	Truncation
	c.1078dupG	p.Asp360fs	1	4.2	4	Truncation

	c.207delG	p.Lys70fs	1	4.2	4	Truncation
	c.225_227delTCT	p.Leu77del	1	4.2	3	Based on structural analysis[d]
	c.364A>C	p.Thr122Pro	1	4.2	3	Based on structural analysis[d]
	c.790G>C	p.Ala264Pro	1	4.2	Undetermined	

[a]Taylor EM, Broughton BC, Botta E, *et al*. Xeroderma pigmentosum and trichothiodystrophy are associated with different mutations in the XPD (ERCC2) repair/transcription gene. *Proc Natl Acad Sci U S A*. 1997;94(16):8658–8663.

[b]Dubaele S, Santis LP De, Bienstock RJ, *et al*. Basal transcription defect discriminates between xeroderma pigmentosum and trichothiodystrophy in XPD patients. *Mol Cell*. 2003;11(6):1635–1646.

[c]Sabatella M, Theil AF, Ribeiro-Silva C, *et al*. Repair protein persistence at DNA lesions characterizes XPF defect with Cockayne syndrome features. *Nucleic Acids Res*. 2018;46(18):9563–9577.

[d]Biertümpfel C, Zhao Y, Kondo Y, *et al*. Structure and mechanism of human DNA polymerase eta. *Nature*. 2010;465(7301):1044–1048.
